# Opposite effects of Activin type 2 receptor ligands on cardiomyocyte proliferation during development and repair

**DOI:** 10.1038/s41467-017-01950-1

**Published:** 2017-12-01

**Authors:** Deepika Dogra, Suchit Ahuja, Hyun-Taek Kim, S. Javad Rasouli, Didier Y. R. Stainier, Sven Reischauer

**Affiliations:** 1 Department of Developmental Genetics, Max Planck Institute for Heart and Lung Research, 61231 Bad Nauheim, Germany; 2 Department of Biochemistry and Biophysics, University of California San Francisco, San Francisco, 94158 CA USA

## Abstract

Zebrafish regenerate damaged myocardial tissue very effectively. Hence, insights into the molecular networks underlying zebrafish heart regeneration might help develop alternative strategies to restore human cardiac performance. While TGF-β signaling has been implicated in zebrafish cardiac regeneration, the role of its individual ligands remains unclear. Here, we report the opposing expression response during zebrafish heart regeneration of two genes, *mstnb* and *inhbaa*, which encode TGF-β family ligands. Using gain-of-function (GOF) and loss-of-function (LOF) approaches, we show that these ligands mediate inverse effects on cardiac regeneration and specifically on cardiomyocyte (CM) proliferation. Notably, we find that Inhbaa functions as a CM mitogen and that its overexpression leads to accelerated cardiac recovery and scar clearance after injury. In contrast, *mstnb* GOF and *inhbaa* LOF both lead to unresolved scarring after cardiac injury. We further show that Mstnb and Inhbaa inversely control Smad2 and Smad3 transcription factor activities through alternate Activin type 2 receptors.

## Abstract (web summary)

Zebrafish can regenerate damaged myocardial tissue but it is unclear how this is regulated. Here, the authors show that two TGF-β family members, Mstnb and Inhbaa, have opposite effects in regeneration, with *mstnb* overexpression or *inhbaa* loss-of-function causing cardiac scarring after injury.

## Introduction

The adult mammalian heart is incompetent to regenerate damaged muscle tissue post myocardial infarction (MI). Instead, lost myocardium is replaced by a functionally and electrically inert fibrotic scar, resulting in compromised cardiac performance and arrhythmia^1^. As a consequence, MI is a leading cause of death and morbidity worldwide^2^. Several active fields of research are trying to address this problem by developing various regenerative approaches focusing on the engraftment of stem cell-derived CMs into injured hearts^3^, the stimulation of CM proliferation in situ^4^, or the in situ trans-differentiation of fibroblasts into functional CMs^5^.

In contrast to mammals, several other vertebrates, including zebrafish, can regenerate injured or lost myocardial tissue^6^ after multiple types of injury, including ventricular resection^6^, cryoinjury^7^, or genetic CM ablation^8^. Consequently, zebrafish serve as an established model to investigate the process of cardiac regeneration. Lineage tracing experiments have reported that remaining CMs in the vicinity of the injured area undergo dedifferentiation and proliferation to give rise to new CMs that subsequently integrate and functionally couple to the remaining myocardium^9, 10^. Hence, an in-depth understanding of the cellular and molecular processes controlling cardiac regeneration appears instrumental to develop alternative therapeutic strategies.

Various signaling pathways including epidermal growth factor (EGF), bone morphogenetic protein, vascular endothelial growth factor, interleukin 6 class cytokines and others have been implicated in the process of cardiac regeneration^11–13^. Importantly, only Neuregulins (Nrg) and their co-receptor ERBB2 have so far been reported to possess mitogenic activity on CMs, not only after injury, but also on the healthy myocardium of fish and mice^4, 14–17^. Consequently, Nrg is subject to ongoing research to evaluate its therapeutic potential^18^. Members of the transforming growth factor beta (TGF-β) signaling pathway have been implicated in various developmental^19^ and disease conditions^20^, but the role of its numerous components in cardiac regeneration is poorly understood. The vertebrate TGF-β/Activin subfamily (TGF-β family) of ligands is comprised of Activins (INHBA, INHBB), GDFs (Myostatin/GDF8, GDF11) and TGF-β (TGFB1, TGFB2, and TGFB3) which bind Activin type 2 receptors (ACVR2A, ACVR2B, TGFBR2), leading to the recruitment and activation of Activin type 1 receptors (ACVR1B, TGFBR1, ACVR1C). Canonically, this process is followed by phosphorylation of the signal transducers Smad2/3, which bind to Smad4 and translocate to the nucleus, thereby modulating the expression of target genes^21–23^. Several non-canonical TGF-β pathways have also been reported, which involve mitogen-activated protein kinases (MAPK) and phosphatidylinositol-3-kinase/Akt^24^. In the diseased mammalian heart, enhanced TGF-β signaling through upregulation of Myostatin (MSTN)^25^, Inhibin betaA (INHBA)^26^, and TGF-β^27^, stimulates hypertrophy, fibrosis, apoptosis, and endothelial–mesenchymal transition^28, 29^. Moreover, global myocardial inhibition of TGF-β signaling through CM-specific *Tgfbr2* deletion reduces pathological remodeling in response to sustained pressure overload^30^. In zebrafish, TGF-β signaling is essential for heart regeneration as chemical inhibition of Activin type 1 receptors suppresses CM proliferation and compromises overall cardiac regeneration^31^. MSTN, a well-known negative regulator of skeletal muscle growth^32, 33^, has been implicated in the development of cardiac hypertrophy in mammals^34, 35^. Further, the absence of MSTN enhances murine skeletal muscle regeneration^36^, and a recently developed monoclonal antibody against MSTN shows therapeutic potential in the treatment of skeletal muscle atrophy^37^. *inhba*, also known as *activin betaA*, promotes wound closure by regulating c-Jun phosphorylation and blastema proliferation during zebrafish fin regeneration^38^. In sum, the role of TGF-β signaling and its various ligands during heart regeneration and pathology remains unclear. Using gene expression profiling, we identified the opposing expression response of *mstnb* and *inhbaa* to cardiac cryoinjury, calling for a detailed investigation of their specific roles during cardiac regeneration.

Here we show that these two TGF-β family ligands antagonize one another during zebrafish cardiac regeneration. While *mstnb* is robustly and continuously downregulated after cryoinjury in the adult zebrafish heart, *inhbaa* is upregulated. Loss of *mstnb* function and activation of *inhbaa* expression are both beneficial for CM proliferation and lead to enhanced cardiac regeneration. Notably, the overexpression (OE) of *inhbaa* alone is sufficient to induce CM proliferation independently of the well-known Nrg–ErbB signaling pathway. Furthermore, we show that Mstnb and Inhbaa function through alternate receptor complexes to control the activities of the signal transducers, Smad2 and Smad3, and regulate CM proliferation during development.

## Results

### *mstnb* and *inhbaa* expression show opposing response to injury

**Fig. 1:**
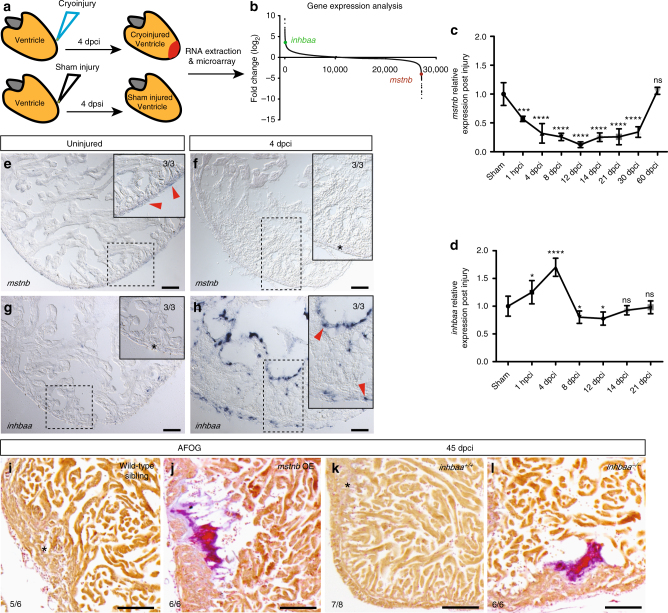
*mstnb* and *inhbaa* have opposing response and functions during zebrafish cardiac regeneration. **a** Schematic representation of injury and sample preparation for microarray analysis (*n* = 12 hearts). **b** Averaged transcriptional gene expression changes post cryoinjury as assessed by microarray analysis (*inhbaa* and *mstnb* indicated). **c**, **d** Temporal RT-qPCR analysis for *mstnb* and *inhbaa* expression post cryoinjury (*n* = 2 × 3 cardiac ventricles assessed as two biological and two technical replicates for each time point, data are mean ± s.e.m., ns: no significant changes observed, *P ≤ 0.05, ***P ≤ 0.001, and ****P ≤ 0.0001—Student’s *t* test, two-tailed). **e**–**h** In situ hybridization for *mstnb* and *inhbaa* expression on uninjured and 4 dpci adult zebrafish heart sections. Higher magnifications of dashed boxes in **e**–**h** are shown in upper right corners. RNA probe signal is indicated by red arrowheads and the absence of signal is indicated by asterisks. The numerators indicate the number of hearts with a particular pattern of signal, and the denominators the total number of hearts analyzed. **i**–**l** AFOG staining of sections from wild-type sibling, *mstnb* OE, *inhbaa* ^*+/+*^, and *inhbaa* ^*−/−*^ cryoinjured hearts at 45 dpci. Healthy myocardium in orange, fibrin in red, collagen in blue. Asterisks indicate the regions of resolved scarring. The numerators indicate the number of hearts with a particular pattern of scarring, and the denominators the total number of hearts analyzed. Scale bars: in situ hybridization images, 50 µm; AFOG staining images, 100 µm

In order to identify genes differentially regulated during adult zebrafish heart regeneration, we performed microarray-based expression profiling of whole hearts 4 days post sham injury (dpsi) vs. 4 days post cryoinjury (dpci) (Fig. 1a). Interestingly, we detected opposing expression response of two genes, *mstnb* and *inhbaa*, encoding TGF-β family ligands (Fig. 1b; raw data have been deposited in the NCBI-Gene Expression Omnibus Website—GSE89259). To obtain more detailed temporal expression data, we quantified *mstnb* and *inhbaa* expression in sham and cryoinjured ventricles at different time points post injury using real-time quantitative PCR (RT-qPCR). Consistent with our microarray data, we observed a rapid reduction of *mstnb* transcript levels in regenerating ventricles compared to sham injury, as early as 1 h post cryoinjury (hpci) (Fig. 1c). Interestingly, *mstnb* expression did not return to basal levels before 60 dpci (Fig. 1c), when regeneration was completed^7^, suggesting that a continuous reduction of *mstnb* expression is important for cardiac regeneration. On the contrary, *inhbaa* showed rapid upregulation, peaking at 4 dpci (Fig. 1d); however, by 8 dpci *inhbaa* expression had returned to sham levels (Fig. 1d), indicating a principal role for *inhbaa* during the early phase of cardiac regeneration. To complement the temporal expression data, we analyzed the spatio-temporal pattern of *mstnb* and *inhbaa* expression by in situ hybridization on sections. Interestingly, *mstnb* expression was strongest in the ventricular wall of uninjured hearts (Fig. 1e), and it was reduced below detection levels in 4 dpci samples (Fig. 1f). We also quantified *mstnb* expression levels in uninjured adult hearts by RT-qPCR on laser micro dissected (LMD) tissues and observed higher levels in the ventricular wall over trabecular tissues (Supplementary Fig. 1a). In contrast, no expression of *inhbaa* could be detected in uninjured hearts (Fig. 1g), while it was prominent proximal to the injury site at 4 dpci (Fig. 1h), consistent with published data based on RNA tomography^11^. To complete our assessment, we examined other TGF-β/Activin ligands and their receptors (refer to Supplementary Table 1 for gene names). Even at a later time point (6 dpci), *inhbaa* and its paralogs *inhbb* and *tgfb3* are the only significantly induced TGF-β family ligand encoding genes, with *inhbaa* showing the most robust upregulation (Supplementary Fig. 1b, c). Taken together, the expression patterns of *inhbaa* and *mstnb* clearly show different spatio-temporal changes during cardiac regeneration.

### *mstnb* GOF and *inhbaa* LOF lead to unresolved scarring

Following our observation that the expression of *mstnb* decreases during cardiac regeneration, we wanted to analyze the effect of sustained *mstnb* expression in regenerating hearts. Hence, we generated a transgenic zebrafish line for CM-specific constitutive OE of *mstnb*, *Tg(myl7:mstnb-2A-H2B-EGFP)* (*mstnb* OE hereafter) (Supplementary Fig. 1d–f). As assessed by RT-qPCR, *mstnb* transcript levels are highly increased in our transgenic *mstnb* OE line compared to non-transgenic siblings (Supplementary Fig. 1g). The gross morphology of adult *mstnb* OE fish and the morphology of their hearts however appear unaffected (Supplementary Fig. 1h–k), indicating that cardiac-specific *mstnb* OE does not affect the development or growth of the zebrafish heart, unlike in mouse hearts, where its OE is reported to cause interstitial fibrosis with compromised cardiac output^39^.

Next, to determine the role of *inhbaa* during cardiac regeneration, we generated a mutant allele using transcription activator-like effector nuclease (TALEN)-induced mutagenesis. A TALEN targeting the TGF-β propeptide domain was designed (Supplementary Fig. 1l) and a 17 bp deletion allele, *inhbaa* ^*bns37*^ (Supplementary Fig. 1m), which is predicted to encode a truncated protein (Supplementary Fig. 1n) was recovered. In addition, *inhbaa* transcript levels were found to be significantly reduced in *inhbaa* ^*−/−*^ compared to *inhbaa* ^*+/+*^ siblings as shown by RT-qPCR (Supplementary Fig. 1o), suggesting active mRNA degradation. Similar to *mstnb* OE animals, *inhbaa* ^*−/−*^ zebrafish do not exhibit any gross morphological defects and their hearts appear indistinguishable from those of wild-type siblings (Supplementary Fig. 1p–s), indicating that *inhbaa* does not play a critical role during zebrafish development.

The zebrafish heart responds to cardiac injury with the formation of a transient fibrotic scar, which is progressively replaced by newly formed healthy myocardium within two months^7^. To assess any possible defect in the process of cardiac regeneration, we tested for scar resolution in cryoinjured *mstnb* OE and *inhbaa* ^*−/−*^ hearts at 45 dpci. By performing acid fuchsin orange G (AFOG) staining on sections, we observed that 45 dpci *mstnb* OE and *inhbaa* ^*−/−*^ hearts were unable to resolve their scar, in contrast to wild-type siblings (Fig. 1i–l) of the same regenerative stage. These data show that both *mstnb* GOF and *inhbaa* LOF interfere with cardiac regeneration and consequently cause reduced scar clearance.

### *mstnb* GOF and *inhbaa* LOF impair CM proliferation post injury

**Fig. 2:**
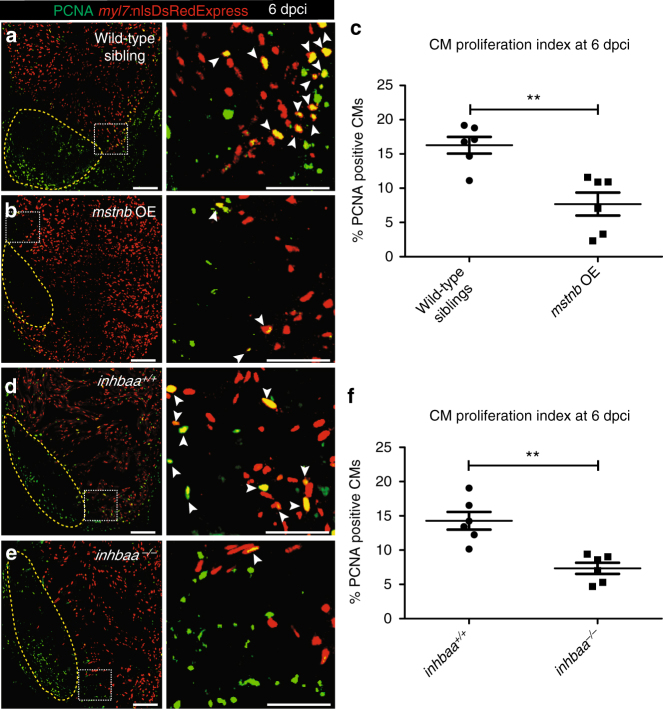
*mstnb* GOF and *inhbaa* LOF suppress CM proliferation during cardiac regeneration. **a**, **b** Sections of wild-type sibling and *mstnb* OE cryoinjured hearts in *Tg(myl7:nlsDsRedExpress)* background at 6 dpci; α-DsRed (red), PCNA (green). Yellow dotted regions delineate the injured area. Higher magnifications of dashed boxes in **a**, **b** are shown on right side. White arrowheads point to proliferating CMs (PCNA^+^/DsRed^+^). **c** Quantification of CM proliferation in wild-type sibling (*n* = 6) and *mstnb* OE (*n* = 6) cryoinjured hearts in the 100 µm region adjacent to the injured area at 6 dpci. **d**, **e** Sections of *inhbaa* ^*+/+*^ and *inhbaa* ^*−/−*^ cryoinjured hearts in *Tg(myl7:nlsDsRedExpress)* background at 6 dpci; α-DsRed (red), PCNA (green). Higher magnifications of dashed boxes in **d**, **e** are shown on right side. **f** Quantification of CM proliferation in *inhbaa* ^*+/+*^ (*n* = 6) and *inhbaa* ^*−/−*^ (*n* = 6) cryoinjured hearts in the 100 µm region adjacent to the injured area at 6 dpci. All cell counts were performed on three sections from each heart. Each data point on dot plot represents one heart (data are mean ± s.e.m., **P ≤ 0.01—Student’s *t* test, two-tailed). Scale bars: heart sections, 100 µm; higher magnifications, 50 µm

Cardiac regeneration relies on the dedifferentiation and cell cycle re-entry of the spared CMs^9^. To test whether *inhbaa* deficiency or *mstnb* OE modulate CM dedifferentiation, we assessed the expression of embryonic cardiac myosin heavy chain (embCMHC), a marker of dedifferentiated CMs^40^, at 6 dpci. We could not observe any obvious differences in embCMHC expression in *mstnb* OE or *inhbaa* ^*−/−*^ hearts compared to their wild-type siblings (Supplementary Fig. 2a–d). Next, we tested whether CM proliferation and cell cycle re-entry was affected in *mstnb* OE and *inhbaa* ^*−/−*^ fish at 6 dpci. We used *Tg(myl7:nlsDsRedExpress)* fish and performed immunostaining for DsRed and PCNA (cell cycle stage marker), and quantified CM proliferation near the injured area. We observed a 53% (±13% s.e.m.) decrease in CM proliferation in *mstnb* OE compared to control (Fig. 2a–c), suggesting that *mstnb* has a significant inhibitory effect on CM proliferation during cardiac regeneration. Similarly, we observed a 49% (±11.5% s.e.m.) reduction in CM proliferation in *inhbaa* ^*−/−*^ compared to *inhbaa* ^*+/+*^ animals (Fig. 2d–f), indicating that *inhbaa* is instrumental for CM proliferation in the regenerating heart. Taken together, these results show that *mstnb* GOF and *inhbaa* LOF negatively affect CM proliferation, indicating that these two TGF-β family ligands have opposite functions during cardiac regeneration.

### *mstnb* LOF and *inhbaa* GOF promote CM proliferation

**Fig. 3:**
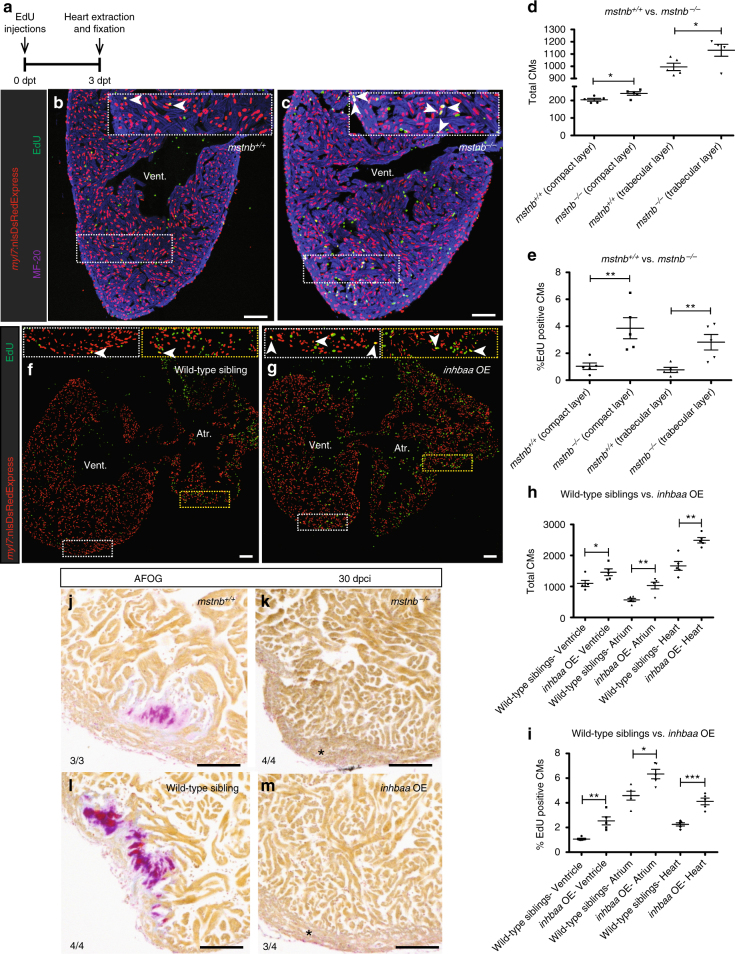
*mstnb* LOF and *inhbaa* GOF positively affect physiological CM proliferation and cardiac regeneration. **a** Experimental setup of EdU treatment, followed by heart extraction and fixation. **b**, **c** Sections of *mstnb* ^*+/+*^ and *mstnb* ^*−/−*^ adult hearts in *Tg(myl7:nlsDsRedExpress)* background; α-DsRed (red), MF-20 (blue), EdU (green). Higher magnifications of dashed boxes in **b**, **c** are shown in upper right corners. White arrowheads point to EdU^*+*^/DsRed^*+*^ CMs. **d**,** e** Quantification of total CMs (DsRed^+^) and EdU incorporating CMs (EdU^*+*^/DsRed^*+*^) in the compact and trabecular layers of *mstnb* ^*+/+*^ (*n* = 5) and *mstnb* ^*−/−*^ (*n* = 5) ventricles. **f**,** g** Sections of wild-type sibling and *inhbaa* OE adult hearts in *Tg(myl7:nlsDsRedExpress)* background; α-DsRed (red), EdU (green). Higher magnifications of dashed boxes in **f**, **g** are shown in upper left and upper right corners. **h**,** i** Quantification of total CMs (DsRed^*+*^) and EdU incorporating CMs (EdU^*+*^/DsRed^*+*^) in wild-type sibling (*n* = 5) and *inhbaa* OE (*n* = 5) hearts. All cell counts were performed on three sections from each heart. Each data point on dot plot represents one heart (data are mean ± s.e.m., *P ≤ 0.05, **P ≤ 0.01, ***P ≤ 0.001—Student’s *t* test, two-tailed). **j**–**m** AFOG staining of sections from *mstnb* ^*+/+*^ (*n* = 3), *mstnb* ^*−/−*^ (*n* = 4), wild-type sibling (*n* = 4), and *inhbaa* OE (*n* = 4) cryoinjured hearts at 30 dpci. Asterisks indicate the regions of resolved scarring. The numerators indicate the number of hearts with a particular pattern of scarring, and the denominators the total number of hearts analyzed. Scale bars, 100 µm. dpt, days post treatment; vent., ventricle; atr., atrium

Further, as a complementary approach, we examined the effects of *mstnb* LOF and *inhbaa* GOF on cardiac development and regeneration. In order to analyze the effect of the loss of *mstnb*, we generated *mstnb* mutant fish using a TALEN targeting the region after the signal peptide domain (Supplementary Fig. 3a) and recovered a 10 bp deletion allele, *mstnb* ^*bns5*^ (Supplementary Fig. 3b), which is predicted to encode a truncated protein (Supplementary Fig. 3c). According to our RT-qPCR data, *mstnb* transcript levels are significantly reduced in *mstnb* ^*−/−*^ compared to *mstnb* ^*+/+*^ (Supplementary Fig. 3d), suggesting mRNA decay. As previously reported in other species^32, 33^, adult *mstnb* ^*−/−*^ fish appear hypermuscular, suggesting a conserved function of Mstn in skeletal muscle growth. Interestingly, we also observed increased heart size in *mstnb* ^*−/−*^ with a thickened ventricular wall (Supplementary Fig. 3e–j, l), the principal expression domain of *mstnb* as detailed previously. Notably, the eye size remained unaffected in *mstnb* ^*−/−*^ compared to wild-type siblings (Supplementary Fig. 3k). To better understand whether the increase in ventricular wall thickness was a consequence of increased CM proliferation in the compact layer, we injected EdU in adult *Tg(myl7:nlsDsRedExpress)* fish and performed immunostainings for DsRed and CM-specific myosin heavy chain (MF-20), followed by EdU labeling on cardiac sections (Fig. 3a). We quantified the total number of CMs as well as EdU incorporation in CMs in the compact and trabecular layers of the ventricle. Notably, hearts from *mstnb* ^*−/−*^ animals showed a significant increase compared to siblings in the total number of CMs and the number of EdU incorporating CMs, in both the wall and the trabeculae of the ventricle (Fig. 3b–e). Our results therefore suggest that *mstnb* LOF promotes CM proliferation, leading to cardiac hyperplasia in zebrafish, unlike in *Mstn*-knockout mice which respond by cardiac hypertrophy^34, 35^.

Next, we generated a transgenic zebrafish line for CM-specific constitutive OE of *inhbaa*, *Tg(myl7:inhbaa-2A-H2B-EGFP)* (*inhbaa* OE hereafter) (Supplementary Fig. 4a–c), resulting in strongly increased *inhbaa* transcript levels compared to control (Supplementary Fig. 4d). The majority of adult (3–6 months post fertilization (mpf)) *inhbaa* OE fish appear morphologically normal when compared to non-transgenic fish; however, the *inhbaa* OE hearts are significantly enlarged and show dense trabeculation in both chambers, whereas the eye size is unaffected (Supplementary Fig. 4e–k). During late adult stages (>6 mpf), we observed ~20% of the *inhbaa* OE fish developing pericardial edema with abnormally enlarged atria, indicating symptoms of a failing heart due to prolonged *inhbaa* OE. Further, to investigate the causality of cardiac enlargement and hypertrabeculation observed in *inhbaa* OE hearts of young adults, we performed EdU injections in adult *Tg(myl7:nlsDsRedExpress)* fish and immunostained for DsRed, followed by EdU labeling (Fig. 3a). Quantification of total number of CMs as well as EdU incorporation in CMs was performed for both chambers. We detected a significant increase in the total number of CMs, as well as in the number of EdU incorporating CMs in *inhbaa* OE hearts compared to control (Fig. 3f–i). Further, we tested whether increased CM proliferation helps these hearts after injury. Thus, we compared *mstnb* ^*−/−*^ and *inhbaa* OE hearts with their siblings at 30 dpci, a time point at which wild-type cryoinjured hearts still retain scarring^7^. By performing AFOG staining on sections, we observed that 30 dpci *mstnb* ^*−/−*^ and *inhbaa* OE hearts had completely resolved scars, in contrast to their wild-type siblings (Fig. 3j–m). Taken together, these results consolidate the finding that *mstnb* and *inhbaa* have opposing effects on CM proliferation during regeneration. Intriguingly, *inhbaa* OE and *mstnb* loss-of-function lead to CM hyperplasia, suggesting that *inhbaa* acts as a mitogen during development and repair.

### *inhbaa* OE promotes CM proliferation independent of ErbB2

**Fig. 4:**
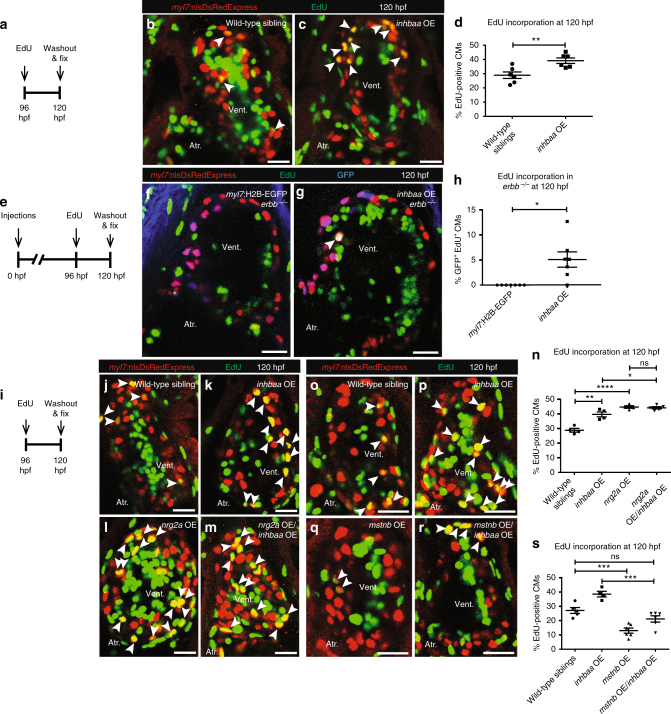
*inhbaa* GOF promotes CM proliferation independently of ErbB2 signaling and competes with m*stnb* GOF. **a** Experimental setup of EdU treatment, followed by fixation. **b**,** c** *Tg(myl7:nlsDsRedExpress)* hearts of wild-type sibling and *inhbaa* OE larvae at 120 hpf; α-DsRed (red), EdU (green). White arrowheads point to proliferating CMs (EdU^*+*^/DsRed^*+*^). **d** Quantification of CM proliferation in wild-type sibling (*n* = 6) and *inhbaa* OE (*n* = 6) ventricles at 120 hpf. **e** Experimental setup of injections, EdU exposure, followed by fixation. **f**,** g** *Tg(myl7:nlsDsRedExpress)* hearts of *myl7:H2B-EGFP* and *myl7:inhbaa-2A-H2B-EGFP(inhbaa OE)* injected *erbb* ^*−/−*^ larvae at 120 hpf; α-DsRed (red), α-GFP (blue), EdU (green). White arrowheads point to proliferating CMs (EdU^*+*^/DsRed^*+*^/GFP^*+*^). **h** Quantification of CM proliferation in *myl7:H2B-EGFP* (*n* = 7) and *inhbaa* OE (*n* = 7) injected ventricles at 120 hpf. **i** Experimental setup of EdU treatment, followed by fixation. **j**–**m** *Tg(myl7:nlsDsRedExpress)* hearts of wild-type sibling, *inhbaa* OE, *nrg2a* OE, and *nrg2a* OE/*inhbaa* OE larvae at 120 hpf; α-DsRed (red), EdU (green). White arrowheads point to proliferating CMs (EdU^*+*^/DsRed^*+*^). **n** Quantification of CM proliferation in wild-type sibling (*n* = 4), *inhbaa* OE (*n* = 4), *nrg2a* OE (*n* = 4), and *nrg2a* OE/*inhbaa* OE (*n* = 5) ventricles at 120 hpf. **o**–**r** *Tg(myl7:nlsDsRedExpress)* hearts of wild-type sibling, *inhbaa* OE, *mstnb* OE, and *mstnb* OE/*inhbaa* OE larvae at 120 hpf; α-DsRed (red), EdU (green). **s** Quantification of CM proliferation in wild-type sibling (*n* = 5), *inhbaa* OE (*n* = 4), *mstnb* OE (*n* = 6), and *mstnb* OE/*inhbaa* OE (*n* = 6) ventricles at 120 hpf. All cell counts were performed on non-overlapping confocal planes (thickness, 1 µm) (data are mean ± s.e.m., ns: no significant changes observed, *P ≤ 0.05, **P ≤ 0.01, ***P ≤ 0.001 and ****P ≤ 0.0001—Student’s *t* test, two-tailed). Scale bars, 20 µm. vent., ventricle; atr., atrium

To further investigate the pro-mitogenic effect of *inhbaa* OE on CMs, we analyzed CM proliferation in larval hearts at 120 h post fertilization (hpf). CM labeling was achieved using the *Tg(myl7:nlsDsRedExpress)* background, which again was combined with an EdU incorporation assay, followed by immunostaining for DsRed (Fig. 4a). We observed an increase of 35% (±11% s.e.m.) in the number of EdU incorporating CMs in *inhbaa* OE larvae compared to control (Fig. 4b–d), indicating that *inhbaa* OE also promotes CM proliferation at early stages. We next wanted to analyze whether the effects of *inhbaa* OE on CM proliferation depended on the Nrg–ErbB signaling pathway, a well-known regulator of CM proliferation^14–16^. Thus, we injected *myl7:inhbaa-2A-H2B-EGFP* and *myl7:H2B-EGFP* plasmid DNA in embryos from *erbb2* ^*st61*^ heterozygote intercrosses and performed EdU incorporation analysis (Fig. 4e), followed by genotyping. Examining GFP^+^ CMs in *erbb2* ^*st61*^ homozygous mutants at 120 hpf, we found that a significant amount of *erbb* ^*−/−*^ CMs expressing *inhbaa* OE were EdU^+^ while *erbb* ^*−/−*^ CMs expressing GFP alone did not show any signs of EdU incorporation (Fig. 4f–h). Next, we treated *inhbaa* OE larvae and wild-type siblings with the established ErbB2 inhibitor PD168393 and analyzed CM proliferation (Supplementary Fig. 5a). We observed that pharmacological inhibition of ErbB2 significantly reduced CM proliferation, as described previously^16^, and interestingly, that *inhbaa* OE was able to rescue this effect. We found that *inhbaa* OE induces an increase of 77.5% (±18% s.e.m.) in the number of EdU incorporating CMs compared to wild-type siblings under ErbB2-blocking conditions (Supplementary Fig. 5b–d).

As recently reported, in zebrafish, Nrg2a signals through ErbB2 to induce CM trabeculation and proliferation^17^. We were interested to compare the effects of *inhbaa* and *nrg2a* OE, and thus decided to test whether the OE of both ligands resulted in an additive effect on CM proliferation. Thus, by crossing *inhbaa* OE and *Tg(myl7:nrg2a-p2a-tdTomato)* (*nrg2a* OE hereafter) fish, we analyzed CM proliferation in 120 hpf *Tg(myl7:nlsDsRedExpress)* larvae, by performing immunostaining for DsRed, followed by EdU labeling (Fig. 4i) and genotyping. As expected from our before-mentioned observations and recently published work^17^, there was an increase of 38% (±7.5% s.e.m.) and 55% (±8% s.e.m.) in the number of EdU incorporating CMs in *inhbaa* OE and *nrg2a* OE larvae, respectively (Fig. 4j–l, n). However, we did not observe any additive effects from overexpressing both ligands, which resulted in a 53% (±6% s.e.m.) increase in the number of EdU incorporating CMs, similar to *nrg2a* OE alone (Fig. 4m, n). This result might indicate that CM proliferation reaches its maximum by *nrg2a* OE alone, as supported by the minimal variability across the assessed samples, or an interference of Inhbaa-mediated proliferation by ErbB signaling. Altogether, these data show that Inhbaa can promote CM proliferation during development and without the need of regenerative stimuli, and that the mitogenic activity of Inhbaa on CMs acts independently of ErbB2 receptor activity.

### *inhbaa* and *mstnb* compete to regulate CM proliferation

Next, to investigate whether *mstnb* and *inhbaa* collaboratively regulate CM proliferation, we crossed our *inhbaa* OE line with the *mstnb* OE line in presence of the CM marking *Tg(myl7:nlsDsRedExpress)* background. Measuring EdU incorporation (Fig. 4i) followed by genotyping, we found that *mstnb* OE reduces myocardial EdU incorporation induced by *inhbaa* OE back to wild-type levels (Fig. 4o–s). Hence, our results suggest that Mstnb and Inhbaa compete in controlling CM cell cycle progression.

### *mstnb* and *inhbaa* overexpression activate distinct Smads

**Fig. 5:**
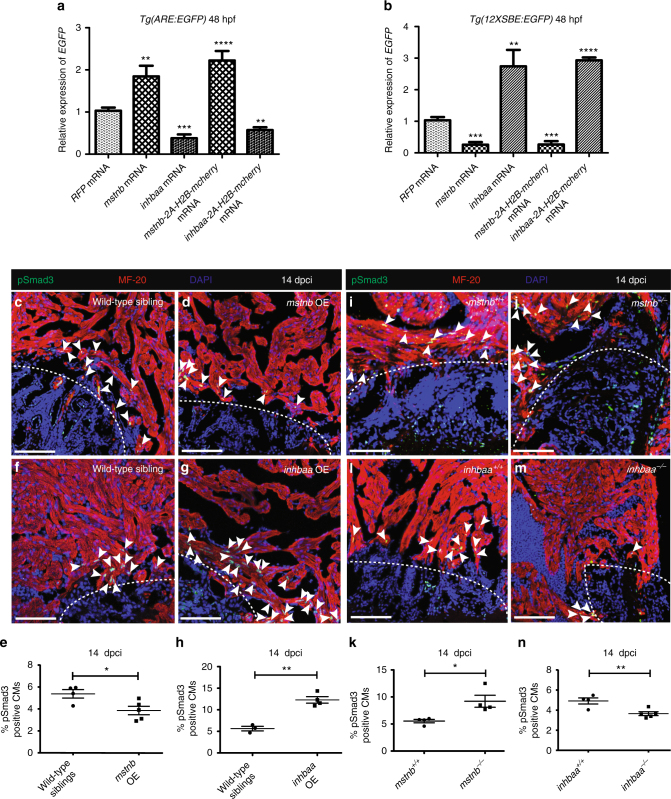
*mstnb* and *inhbaa* inversely regulate the activities of Smad2 and Smad3 response elements, as well as Smad3 phosphorylation. **a** RT-qPCR analysis for relative *EGFP* mRNA expression in 48 hpf *Tg(ARE:EGFP)* embryos injected with *mstnb*, *inhbaa*, *mstnb-2A-H2B-mcherry,* and *inhbaa-2A-H2B-mcherry* mRNA compared to *RFP* mRNA injected (*n* = 2 × 10 embryos assessed as 2 biological and 2 technical replicates). **b** RT-qPCR analysis for relative *EGFP* mRNA expression in 48 hpf *Tg(12XSBE:EGFP)* embryos injected with *mstnb*, *inhbaa*, *mstnb-2A-H2B-mcherry*, and *inhbaa-2A-H2B-mcherry* mRNA compared to *RFP* mRNA injected (*n* = 2 × 10 embryos assessed as two biological and two technical replicates). **c**, **d** Sections of wild-type sibling and *mstnb* OE cryoinjured hearts at 14 dpci; pSmad3 (green), α-MF-20 (red), DAPI (blue). White dotted regions delineate the injured area. White arrowheads point to pSmad3^+^ CMs near the injured area. **e** Quantification of pSmad3^+^ CMs in wild-type sibling (*n* = 4) and *mstnb* OE (*n* = 5) cryoinjured hearts in the 100 µm region adjacent to the injured area at 14 dpci. **f**, **g** Sections of wild-type sibling and *inhbaa* OE cryoinjured hearts at 14 dpci; pSmad3 (green), α-MF-20 (red), DAPI (blue). **h** Quantification of pSmad3^+^ CMs in wild-type sibling (*n* = 3) and *inhbaa* OE (*n* = 4) cryoinjured hearts in the 100 µm region adjacent to the injured area at 14 dpci. **i**, **j** Sections of *mstnb* ^*+/+*^ and *mstnb* ^*−/−*^ cryoinjured hearts at 14 dpci; pSmad3 (green), α-MF-20 (red), DAPI (blue). **k** Quantification of pSmad3^+^ CMs in *mstnb* ^*+/+*^ (*n* = 4) and *mstnb* ^*−/−*^ (*n* = 4) cryoinjured hearts in the 100 µm region adjacent to the injured area at 14 dpci. **l**, **m** Sections of *inhbaa* ^*+/+*^ and *inhbaa* ^*−/−*^ cryoinjured hearts at 14 dpci; pSmad3 (green), α-MF-20 (red), DAPI (blue). **n** Quantification of pSmad3^+^ CMs in *inhbaa* ^*+/+*^ (*n* = 4) and *inhbaa* ^*−/−*^ (*n* = 5) cryoinjured hearts in the 100 µm region adjacent to the injured area at 14 dpci. All cell counts were performed on three sections from each heart. Each data point on dot plot represents one heart (data are mean ± s.e.m., *P ≤ 0.05, **P ≤ 0.01, ***P ≤ 0.001 and ****P ≤ 0.0001—Student’s *t* test, two-tailed). Scale bars, 100 µm

We further aimed to decipher the molecular mechanisms underlying the differential regulation of CM proliferation and therefore, cardiac regeneration by *mstnb* and *inhbaa*. Myostatin and Activin have been reported to act through the TGF-β signaling cascade, leading to the phosphorylation of Smad2 and Smad3^21, 22, 41^. Smad2, along with Smad4 and transcription factors such as FAST1/2, binds to the activin response elements (ARE) present in the promoter regions of target genes^42–44^. Similarly, Smad3 binds to the Smad-binding elements (SBE) present in the promoter region of target genes^45, 46^. To identify potentially distinct transcriptional target genes of Mstnb and Inhbaa signaling, we used the published Smad2 reporter line, *Tg(ARE:EGFP)* ^47^ and Smad3 reporter line, *Tg(12XSBE:EGFP)* ^48^. After injecting *mstnb-2A-H2B-mcherry* and *inhbaa-2A-H2B-mcherry* mRNA in *Tg(ARE:EGFP)* and *Tg(12XSBE:EGFP)* embryos at the one-cell stage, we quantified *EGFP* mRNA expression at 48 hpf by RT-qPCR. Our results show a robust induction of Smad2 reporter expression and a downregulation of Smad3 reporter expression after *mstnb-2A-H2B-mcherry* mRNA injections (Fig. 5a, b). Conversely, we found that *inhbaa-2A-H2B-mcherry* mRNA injections induced Smad3 reporter activity and suppressed *EGFP* mRNA expression in the Smad2 reporter system (Fig. 5a, b). To make sure that our P2A-labeling strategy did not interfere with protein function and overall specificity, the aforementioned experiments were also performed with non-tagged wild-type versions leading to results identical in magnitude and specificity (Fig. 5a, b). This differential regulation of the activities of distinct Smad responsive elements by *mstnb* and *inhbaa* might account for their different influence on CM proliferation.

To further validate these results, we investigated the transcriptional response of several known TGF-β targets in response to *inhbaa* or *mstnb* OE. Several TGF-β target genes have been reported to be specific targets of Smad2, including *Goosecoid (Gsc)* ^44^ and *Mix.2* ^43^ or Smad3, including *JunB* ^45^ and *Plasminogen activator inhibitor-1(PAI-1)* ^46^. We thus analyzed the expression of these target genes (along with their paralogs) in 48 hpf *mstnb-2A-H2B-mcherry* mRNA and *inhbaa-2A-H2B-mcherry* mRNA injected embryos, by RT-qPCR. Interestingly, we found an upregulation of Smad2 target gene expression by *mstnb* OE but a downregulation of their expression after *inhbaa* OE (Supplementary Fig. 6a–d). Conversely, we observed an upregulation of Smad3 target gene expression by *inhbaa* OE but a downregulation of their expression after *mstnb* OE (Supplementary Fig. 6e–l). We further tested whether *mstnb* and *inhbaa* OE was able to regulate the expression of these Smad target genes in the injured adult heart. By performing RT-qPCR at 4 dpci, we found that the expression of Smad2 target genes was induced in *mstnb* OE hearts, whereas either no significant effect or a transcriptional downregulation was observed in *inhbaa* OE hearts (Supplementary Fig. 7a–d). Conversely, *inhbaa* OE was able to induce the expression of Smad3 target genes in regenerating hearts, whereas their expression was either unchanged or downregulated after *mstnb* OE (Supplementary Fig. 7e–l). These results further suggest that Smad2 and Smad3 activities are inversely regulated by *mstnb* and *inhbaa*.

### *mstnb* and *inhbaa* inversely regulate Smad3 phosphorylation

Smad3-dependent TGF-β signaling has previously been linked to cardiac regeneration as Activin type 1 receptor inhibition caused a reduction in the number of pSmad3^+^ CMs and ultimately blocked cardiac regeneration^31^. Additionally, by using the same chemical inhibitor, it has been shown that the inhibition of TGF-β signaling negatively affects CM proliferation in zebrafish^49^. Thus, in order to test whether *mstnb* and *inhbaa* affect the phosphorylation of myocardial Smad3 during cardiac regeneration, we performed immunostainings for pSmad3 and MF-20 using a DAPI counterstain at 14 dpci, followed by quantification of pSmad3^+^ CMs proximal to injury site in the respective gain-of-function and loss-of-function genotypes. Interestingly, we found that OE of *mstnb* inhibits Smad3 phosphorylation and detected a 28% (±10% s.e.m.) decrease in the number of pSmad3^+^ CMs in *mstnb* OE compared to control (Fig. 5c–e). Conversely, OE of *inhbaa *induced myocardial Smad3 phosphorylation, with an increase of 118% (±20% s.e.m.) in the number of pSmad3^+^ CMs compared to control (Fig. 5f–h). Notably, we also observed that *mstnb* ^*−/−*^ hearts show an induction of Smad3 phosphorylation in CMs, with an increase of 66% (±21% s.e.m.) in the number of pSmad3^+^ CMs compared to control (Fig. 5i–k). Further, we detected a decline of 26% (±7.5% s.e.m.) in the number of pSmad3^+^ CMs in *inhbaa* ^*−/−*^ compared to control (Fig. 5l–n), indicating that Inhbaa is important to induce Smad3 phosphorylation during cardiac regeneration. Overall, our data indicate that Mstnb and Inhbaa act antagonistically to one another in controlling Smad3 phosphorylation, an apparently crucial event during cardiac regeneration in zebrafish.

### Smad2 and Smad3 inversely affect CM proliferation

**Fig. 6:**
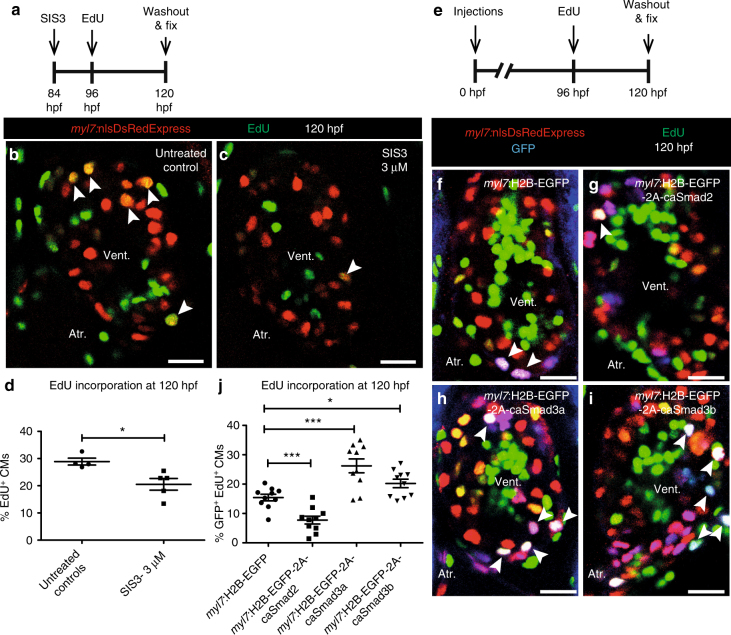
CM proliferation is inversely regulated by Smad2 and Smad3. **a** Experimental setup of SIS3 treatment, followed by EdU treatment and fixation. **b**,** c** *Tg(myl7:nlsDsRedExpress)* hearts of untreated control and 3 µM SIS3-treated larvae at 120 hpf; α-DsRed (red), EdU (green). White arrowheads point to proliferating CMs (EdU^*+*^/DsRed^*+*^). **d** Quantification of CM proliferation in untreated control (*n* = 4) and 3 µM SIS3-treated (*n* = 5) ventricles at 120 hpf. **e** Experimental setup of injections, followed by EdU treatment and fixation. **f**–**i** *Tg(myl7:nlsDsRedExpress)* hearts of *myl7:H2B-EGFP*, *myl7:H2B-EGFP-2A-caSmad2*, *myl7:H2B-EGFP-2A-caSmad3a* and *myl7:H2B-EGFP-2A-caSmad3b* injected larvae at 120 hpf; α-DsRed (red), α-GFP (blue), EdU (green). White arrowheads point to proliferating CMs (EdU^+^/DsRed^+^/GFP ^+^). **j** Quantification of CM proliferation in *myl7:H2B-EGFP* (*n* = 10), *caSmad2* (*n* = 10), *caSmad3a* (*n* = 10), and *caSmad3b* (*n* = 10) injected ventricles at 120 hpf. All cell counts were performed on non-overlapping confocal planes (thickness, 1 µm) (data are mean ± s.e.m., *P ≤ 0.05 and ***P ≤ 0.001—Student’s *t* test, two-tailed). Scale bars, 20 µm. vent., ventricle; atr., atrium

Myocardial Smad3 phosphorylation is induced at the site of injury during cardiac regeneration^31^. Our data from regenerating *mstnb* OE and *inhbaa* ^*−/−*^ hearts show that myocardial Smad3 phosphorylation proximal to the lesion was reduced (Fig. 5c–e, l–n), as was CM proliferation. In order to test the hypothesis that Smad3 phosphorylation is directly linked to CM proliferation, we used a small molecule inhibitor, SIS3, to block TGF-β-mediated Smad3 phosphorylation^50, 51^. By performing RT-qPCR, we first tested whether SIS3 acts as a specific inhibitor of Smad3 phosphorylation, and found that Smad3 target gene expression was downregulated in 72 hpf SIS3-treated hearts (Supplementary Fig. 8h–l), while Smad2 target gene expression remained unchanged (Supplementary Fig. 8e–g). We also analyzed the effect of the established Activin type 1 receptor inhibitor (SB431542) on Smad target gene expression, by performing RT-qPCR in 72 hpf SB431542-treated hearts. We observed a downregulation in the expression of both Smad2 and Smad3 target genes (Supplementary Fig. 8e–l), confirming that this inhibitor blocks the complete TGF-β pathway as reported previously^52^. Next, we treated *Tg(myl7:nlsDsRedExpress)* larvae with SIS3 and analyzed CM proliferation by performing immunostaining for DsRed, followed by EdU labeling at 120 hpf (Fig. 6a). Quantification of EdU incorporation in CMs of larval ventricles revealed that 3 µM SIS3 was sufficient to substantially reduce the number of EdU^+^ CMs (Fig. 6b–d), suggesting that CM proliferation relies on Smad3 phosphorylation.

To further test our model, we analyzed the effects of constitutively active (ca) Smad2, Smad3a and Smad3b on CM proliferation following mosaic OE. For this analysis, we generated constructs expressing ca, phosphomimetic, Smads under the CM-specific *myl7* promoter, namely *Tg(myl7:H2B-EGFP-2A-caSmad2)* (*caSmad2* hereafter), *Tg(myl7:H2B-EGFP-2A-caSmad3a)* (*caSmad3a* hereafter), *Tg(myl7:H2B-EGFP-2A-caSmad3b)* (*caSmad3b* hereafter), and *Tg(myl7:H2B-EGFP)* as control (Supplementary Fig. 8a–d). We first tested whether the *caSmad2, caSmad3a*, and *caSmad3b* constructs were functional and specific, by performing RT-qPCR analysis for Smad2 and Smad3 target gene expression on hearts of 72 hpf larvae obtained from outcrossing the *caSmad2, caSmad3a,* and *caSmad3b* founders. We observed an upregulation in Smad2 target gene expression in *caSmad2* hearts (Supplementary Fig. 8e–g), and an upregulation in Smad3 target gene expression in *caSmad3a* and *caSmad3b* hearts (Supplementary Fig. 8h–l). After injection of the constructs at the one-cell stage, we analyzed CM proliferation by performing immunostaining for GFP and DsRed, followed by EdU labeling in 120 hpf *Tg(myl7:nlsDsRedExpress)* larvae (Fig. 6e). Comparing GFP^+^ CMs across all constructs, we found that CMs expressing *caSmad2* showed a 50% (±12% s.e.m.) reduction in EdU incorporation compared to CMs expressing GFP alone, whereas both *caSmad3a* and *caSmad3b* expression resulted in a 70% (±18% s.e.m.) and 31% (±12% s.e.m.) increase in EdU incorporation, respectively (Fig. 6f–j). These results clearly indicate that Smad2 and Smad3 inversely regulate CM proliferation in zebrafish and could potentially explain how different TGF-β family ligands can have opposite effects during regeneration.

### Mstnb and Inhbaa signal through distinct Activin receptors

**Fig. 7:**
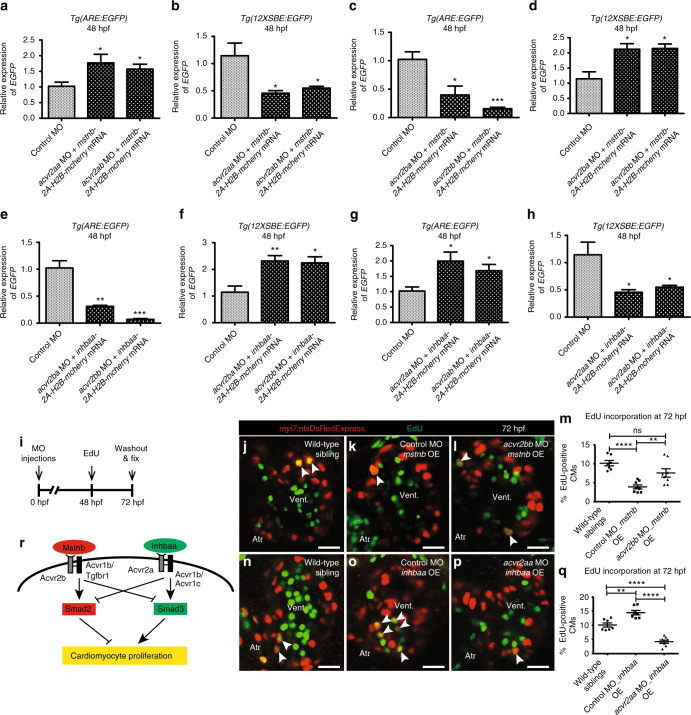
Mstnb and Inhbaa work through distinct Activin type 2 receptors to regulate CM proliferation. **a**–**d** RT-qPCR analysis for relative *EGFP* mRNA expression in 48 hpf *Tg(ARE:EGFP)* and *Tg(12XSBE:EGFP)* embryos injected with *acvr2aa* MO/*mstnb-2A-H2B-mcherry* mRNA, *acvr2ab* MO/*mstnb-2A-H2B-mcherry* mRNA, *acvr2ba* MO/*mstnb-2A-H2B-mcherry* mRNA, and *acvr2bb* MO/*mstnb-2A-H2B-mcherry* mRNA compared to control MO injected (*n* = 2 × 10 embryos assessed as two biological and two technical replicates). **e**–**h** RT-qPCR analysis for relative *EGFP* mRNA expression in 48 hpf *Tg(ARE:EGFP)* and *Tg(12XSBE:EGFP)* embryos injected with *acvr2ba* MO/*inhbaa-2A-H2B-mcherry* mRNA, *acvr2bb* MO/*inhbaa-2A-H2B-mcherry* mRNA, *acvr2aa* MO/*inhbaa-2A-H2B-mcherry* mRNA, and *acvr2ab* MO/*inhbaa-2A-H2B-mcherry* mRNA compared to control MO-injected (*n* = 2 × 10 embryos assessed as two biological and two technical replicates). **i** Experimental setup of injections, followed by EdU treatment and fixation. **j**–**l** *Tg(myl7:nlsDsRedExpress)* hearts of wild-type sibling, control MO-injected *mstnb* OE and *acvr2bb* MO-injected *mstnb* OE larvae at 72 hpf; α-DsRed (red), EdU (green). White arrowheads point to proliferating CMs (EdU^+^/DsRed^+^). **m** Quantification of CM proliferation in wild-type sibling (*n* = 7), control MO-injected *mstnb* OE (*n* = 8) and *acvr2bb* MO-injected *mstnb* OE (*n* = 8) ventricles at 72 hpf. **n**–**p** *Tg(myl7:nlsDsRedExpress)* hearts of wild-type sibling, control MO-injected *inhbaa* OE, and *acvr2aa* MO-injected *inhbaa* OE larvae at 72 hpf; α-DsRed (red), EdU (green). **q** Quantification of CM proliferation in wild-type sibling (*n* = 7), control MO-injected *inhbaa* OE (*n* = 7) and *acvr2aa* MO-injected *inhbaa* OE (*n* = 8) ventricles at 72 hpf. **r** Model of ligand-receptor relationship: Mstnb binds to Acvr2b, leading to the activation of Acvr1b/Tgfbr1, which promotes Smad2 and suppresses Smad3 activation. Inversely, Inhbaa binds to Acvr2a, recruiting Acvr1b/Acvr1c, thereby inducing Smad3 and suppressing Smad2 activation. This process is followed by the differential regulation of CM proliferation by Smad2 and Smad3. All cell counts were performed on non-overlapping confocal planes (thickness, 1 µm) (data are mean ± s.e.m., ns: no significant changes observed, *P ≤ 0.05, **P ≤ 0.01, ***P ≤ 0.001 and ****P ≤ 0.0001—Student’s *t* test, two-tailed). Scale bars, 20 µm. vent., ventricle; atr., atrium

Mstn and Inhba have been described to signal through the same type 2 receptor complex^21, 22, 41^. We wanted to investigate how Mstnb and Inhbaa could function antagonistically if they work through the same signaling cascade. Thus, to better understand the ligand-receptor relationships, we performed combined gene knockdown and OE experiments. We co-injected morpholinos (MOs) for the activin type 2 receptor genes *(acvr2aa, acvr2ab, acvr2ba,* and *acvr2bb)* with *mstnb-2A-H2B-mcherry* mRNA or *inhbaa-2A-H2B-mcherry* mRNA in the Smad2^47^ and Smad3^48^ reporter lines. By performing RT-qPCR for *EGFP* mRNA expression, we found that co-injection of *acvr2a* MOs (*acvr2aa* and *acvr2ab*) along with *mstnb-2A-H2B-mcherry* mRNA did not influence the effect of *mstnb* mRNA injections, since Smad2 reporter expression was induced and Smad3 reporter expression was suppressed compared to control (Fig. 7a, b). In contrast, the co-injection of *acvr2b* MOs *(acvr2ba* and *acvr2bb)* along with *mstnb-2A-H2B-mcherry* mRNA significantly reduced the effect of *mstnb* OE on Smad2 and Smad3 reporter activity (Fig. 7c, d). These results indicate that Mstnb has a preference for binding Acvr2b over Acvr2a, as per previous studies^41, 53^. Next, we tested the relationship between Inhbaa and Acvr2ba and Acvr2bb. We found that the co-injection of *acvr2b* MOs *(acvr2ba* and *acvr2bb)* with *inhbaa-2A-H2B-mcherry* mRNA did not influence the effect of *inhbaa* OE, as Smad2 reporter expression was suppressed and Smad3 reporter expression was induced compared to control (Fig. 7e, f). In contrast, the co-injection of *acvr2a* MOs *(acvr2aa* and *acvr2ab)* along with *inhbaa-2A-H2B-mcherry* mRNA significantly reduced the effect of *inhbaa* OE on Smad2 and Smad3 reporter activity (Fig. 7g, h). These data suggest a previously unreported preference for Inhbaa to signal through Acvr2a over Acvr2b.

Finally, we also analyzed the effect of these receptor knockdowns in the CM EdU incorporation assay using our *mstnb* OE and *inhbaa* OE lines (Fig. 7i). Since its effect on Smad2 reporter expression was more significant compared to *acvr2ba* MO, we injected the *acvr2bb* MO in the *mstnb* OE line and assessed CM EdU incorporation at 72 hpf. We observed a significant increase of 94% (±33% s.e.m.) in CM EdU incorporation in *acvr2bb* MO-injected *mstnb* OE larvae compared to control (Fig. 7j–m). This result is in line with our RT-qPCR data that Acvr2b is a specific receptor for Mstnb, and knocking down *acvr2bb* prevents the suppression of CM proliferation by *mstnb* OE. Next, we injected *acvr2aa* MO in the *inhbaa* OE line and examined CM EdU incorporation at 72 hpf. Here, we observed a significant reduction of 70% (±8% s.e.m.) in CM EdU incorporation in *acvr2aa* MO-injected *inhbaa* OE larvae compared to control (Fig. 7n–q). Again, this result is in line with our RT-qPCR data that Acvr2a is a specific receptor for Inhbaa, and knocking down *acvr2aa* prevents the induction of CM proliferation by *inhbaa* OE. Overall, our results suggest that Mstnb binds to Acvr2b, leading to the activation of Acvr1b/Tgfbr1, which promotes Smad2 and suppresses Smad3 activation. Inversely, Inhbaa binds to Acvr2a, leading to the activation of Acvr1b/Acvr1c, which promotes Smad3 and suppresses Smad2 activation (Fig. 7r).

## Discussion

The role of TGF-β signaling in organ regeneration and pathology remains unclear as seemingly contradictory results have been reported. TGF-β signaling is required for the formation of the wound epithelium and cell proliferation during *Xenopus* tail regeneration^54^, while its loss has been associated with an expansion of progenitor cells in the regenerating mammalian liver^55^. Furthermore, TGF-β signaling has been implicated in the pathogenesis of cardiac remodeling and fibrosis after pressure overload in mammals^30^, while the treatment of adult zebrafish with a TGF-β receptor inhibitor blocked cardiac regeneration^31^. Thus, because of its highly diverse and seemingly contradictory functions across various cell types and organisms, we wanted to dissect the role of different TGF-β family members in cardiac regeneration by performing ligand-specific genetic manipulations.

Here, we have identified the opposing expression response of two TGF-β family ligand encoding genes, *mstnb* and *inhbaa*. From subsequent genetic studies we have revealed their contrasting roles in regulating cardiac regeneration and CM proliferation in zebrafish. Our study shows that expression of *mstnb*, a negative regulator of skeletal muscle growth^32, 33^, is rapidly and continuously downregulated during cardiac regeneration in the adult zebrafish heart. However, this observation contrasts previous findings in mammals which show a rapid and significant upregulation of MSTN post MI^25^. We therefore hypothesized that *mstnb* downregulation in zebrafish following cardiac injury facilitates cardiac regeneration, whereas elevated MSTN levels in the injured mammalian heart could possibly inhibit the process of regeneration. Supporting our hypothesis, we observed that loss of *mstnb* positively affects physiological CM proliferation and cardiac regeneration. Contrarily, *mstnb* OE in CMs led to a significant decline in CM proliferation, reduced regeneration and compromised scar clearance after injury. Overall, our data strongly support that *mstnb* downregulation is important during cardiac regeneration, facilitating CM proliferation.

In contrast, we have found that *inhbaa* expression was upregulated in response to cardiac injury during the early stages of myocardial regeneration. Further, the loss of *inhbaa* strongly correlated with reduced scar clearance and CM proliferation within the proximity of the injury site. Intriguingly, CM-specific *inhbaa* OE not only enhanced cardiac regeneration, but also resulted in cardiomegaly as a consequence of increased CM proliferation and hypertrabeculation, even in the absence of cardiac injury. The cardiac hypertrabeculation phenotype of the *inhbaa* OE fish is reminiscent of that observed in mice mutant for FKBP12^56^, a negative regulator of the TGF-β family^57^, suggesting a conserved function of TGF-β signaling in cardiac development and possibly CM proliferation. Recent reports have identified Nrg as a potent mitogen in fish and mammals^4, 14, 17^. We performed epistasis experiments to test whether *inhbaa-*induced CM proliferation depends upon Nrg–ErbB signaling. These experiments revealed that Inhbaa stimulates CM proliferation independently of ErbB receptor activity, indicating that similar to Nrg, Inhbaa has the potential to induce CM proliferation directly. However, simultaneous OE of *nrg2a* and *inhbaa* did not yield a higher CM proliferative index than OE of *nrg2a* alone. Possibly, stimulation of CM proliferation becomes saturated by *nrg2a* OE alone, as suggested by the minimal variability observed across the different specimens. Alternatively, Nrg2a signaling might interfere with the potential of Inhbaa to promote CM proliferation. Indeed, stimulation of the MAPK signaling cascade by oncogenic mutations in Ras or by EGF receptor stimulation mediates the phosphorylation of specific residues in the linker region of Smad2/3^58^. Phosphorylation of this linker region was shown to inhibit TGF-β-induced C-terminal phosphorylation and nuclear accumulation of Smads, attenuating the transcriptional activation of their target genes^58^. Interestingly, *inhbaa* is upregulated after cardiac injury, both in mammals^26^ and zebrafish, but potentially the timing and levels of its induction do not allow it to induce regeneration in the mammalian heart. In mammals, prolonged upregulation of *INHBA* has been associated with induction of fibrosis post MI, leading to cardiac remodeling and failure^26^. We speculate that a transient upregulation of *INHBA* in injured hearts, as observed in zebrafish, along with a rapid inactivation of *MSTN* could be beneficial to stimulate CM proliferation while prolonged *INHBA* expression has negative consequences. Indeed, several of our *inhbaa* OE animals showed signs of cardiomyopathy and heart failure past 6 months of age, an effect that could be potentiated by the presence of a higher number of myofibroblasts and other non-myocardial cells in the mammalian heart^59^.

Canonically, TGF-β family ligands including Myostatin and Activin signal through Activin type 2 and type 1 receptors leading to the C-terminal phosphorylation of Smad2 and Smad3. Mechanistically, it has been reported that Myostatin has a binding preference for the Activin type 2 receptor Acvr2b compared to Acvr2a^41, 53^. We experimentally validated that not only Mstnb, but also Inhbaa, have different binding affinities for Activin type 2 receptors and inversely modulate the activities of Smad2 and Smad3. Smad2 and Smad3 have highly homologous MH1 and MH2 domains; however, the MH1 domain of Smad2 has 30 extra amino acids preventing its direct binding to DNA, unlike Smad3 which can directly bind to target DNA sequences^60^. These structural differences in Smad2 and Smad3 may account for differences in their functions. Several recent studies have revealed the antagonistic effects of Smad2 and Smad3 during multiple cellular processes such as blastema formation in regeneration, tumor angiogenesis, and neurogenesis^51, 61, 62^. Similarly, our data suggest that constitutively active Smad2 and Smad3 act antagonistically to one another in regulating CM proliferation. Additionally, we identified differential effects of *mstnb* and *inhbaa* OE on myocardial Smad3 phosphorylation in the regenerating heart, suggesting that indeed these two ligands are inversely affecting Smad3 phosphorylation which seemingly mediates CM proliferation during cardiac regeneration. Taken together, we identified opposite functions for the two TGF-β family ligands, Mstnb and Inhbaa, during cardiac regeneration and similarly, for their downstream effectors Smad2 and Smad3. In addition, the Nrg–ErbB independent mitogenic activity of Inhbaa may provide new avenues towards developing alternative strategies in the treatment of patients post MI.

## Methods

### Zebrafish

Procedures involving animals were approved by the veterinary department of the Regional Board of Darmstadt.

### Zebrafish husbandry

All zebrafish husbandry was performed under standard conditions in accordance with institutional (MPG) and national ethical and animal welfare guidelines. The characterized mutant and transgenic lines *erbb2* ^*st61*^ ^63^, *Tg(−0.8myl7:nlsDsRedExpress)hsc4* ^64^, *Tg(myl7:nrg2a202-p2a-tdTomato)bns140* ^17^, *Tg(ARE:EGFP)fci100* ^47^, and *Tg(12XSBE:EGFP)ia16* ^48^ were used in this study.

### Cryoinjury

To perform cryoinjury^7^, adult zebrafish (3–6 mpf) were anaesthetized in 0.016% tricaine and placed on a wet sponge with their ventral side up. An incision was made through the chest to access the heart and a precooled cryoprobe was applied to the ventricular apex till the cryoprobe thawed. Later, the fish were recovered by transferring them to the fresh water.

### Microarray expression profiling

Total RNA was isolated from ~6 mpf sham operated and cryoinjured hearts 4 dpci using Trizol (Life Technologies). Dual color cDNA labeling and hybridization was performed by *MOgene* (commercial service) using the Agilent Zebrafish (V3) 4 × 44 K platform. Microarray raw and normalized data have been submitted to NCBI-GEO under the accession number GSE89259.

### LMD

LMD (LMD-6000, Leica) was performed on adult zebrafish heart cryosections to dissect the wall and trabecular tissues separately for RNA extraction.

### Larval heart extraction

To perform heart extraction^65^, 72 hpf larvae expressing GFP under CM-specific myosin, light chain 7, regulatory (*myl7*) promoter were anesthetized and transferred to 1.5 ml microfuge tube, followed by washing three times with embryo disruption medium (EDM) and resuspension in EDM. Further, 1 ml of EDM containing larvae was drawn into the 19-gauge needle and ejected back into the microfuge tube 30 times at the rate of 1 s per syringe medium. Fragmented larvae were passed through 100 μm nylon mesh and the flow-through was collected in the petri dish. Later, the flow-through was passed through 40 μm nylon mesh. Next, the mesh was inverted and the retained material was washed off with EDM into the petri dish. Intact GFP^+^ hearts were identified under fluorescent light and collected in fresh EDM. These hearts were further pooled in a 1.5 ml microfuge tube, pelleted and the preparations were stored at −80 °C after removing the media. A pool of 30 extracted hearts was used for RNA extraction.

### RT-qPCR

RNA from adult heart ventricles and 48 hpf embryos was extracted using Trizol. RNA from extracted larval hearts and LMD samples was extracted using miRNeasy Micro Kit (Qiagen), and the cDNA prepared (Maxima First Strand cDNA Synthesis Kit for RT-qPCR, Thermo Fisher Scientific) was used to perform RT-qPCR (CFX Connect Real-Time System, Biorad). The primers used for RT-qPCR are listed in Supplementary Table 2. *rpl13* was used as an internal control. The Ct values of the genes in the control samples are listed in Supplementary Table 3.

### Generation of mutants and transgenic zebrafish

*inhbaa* and *mstnb* mutants were generated using TALEN-induced mutagenesis. The TALENs were designed and cloned according to Golden Gate Assembly^66^. The TALEN arms targeting *inhbaa* had the following RVDs: NN NG NN NN NG NN NN NI NN NN HD NI NN NG NN and NN HD NI NN NN NG NN HD NI NN HD NI NG NN NG. The TALEN arms targeting *mstnb* had the following RVDs: NN NN NI NN NI NG NI NG NI NI HD NN NN HD NN HD and HD NN HD NG NG NG HD HD NG HD HD NN NG NN NN HD. The TALEN mRNA were injected in one-cell stage embryos and mutant alleles, *mstnb* ^*bns5*^ and *inhbaa* ^*bns37*^ were recovered by performing High Resolution Melt Analysis, using the following primers: *inhbaa*_F-5′-AGAGCGAGGACGAGGGAG-3′, *inhbaa*_R-5′-GTGTGTGATGTTGGGTCGCT-3′ and *mstnb*_F-5′-GTGTATTAATTGCATGTGGTCCAG-3′, *mstnb*_R-5′-GAACACTGCTCGCTTTCCTC-3′. The F1 heterozygous animals were intercrossed to raise F2 adults, which were used in the experiments.

For generating OE transgenes under the control of *myl7* promoter, the CDS of *mstnb* and *inhbaa* was PCR amplified and fused with a self-cleaving peptide 2A and H2B-EGFP, using Cold Fusion technology (System Biosciences, CA, USA). A total of 18 pg of each of these constructs were co-injected with 20 pg Tol2 mRNA into one-cell stage embryos. The transgenic fish obtained were named as *Tg(myl7:mstnb-2A-H2B-EGFP)bns145* and *Tg(myl7:inhbaa-2A-H2B-EGFP)bns146*. The founders were outcrossed with *Tg(−0.8myl7:nlsDsRedExpress)hsc4* to generate stable lines, which were used in the experiments.

### mRNA overexpression

Full length *mstnb* and *inhbaa* CDS was amplified from cDNA and cloned into pcDNA3.1. *myl7:mstnb-2A-H2B-mcherry* and *myl7:inhbaa-2A-H2B-mcherry* constructs were generated using Cold Fusion technology. For mRNA synthesis, *mstnb-2A-H2B-mcherry* and *inhbaa-2A-H2B-mcherry* CDS was cloned into pcDNA3.1. mRNA was synthesized using the mMESSAGE mMACHINE kit and 100 pg of each mRNA was injected into *Tg(ARE:EGFP)* and *Tg(12XSBE:EGFP)* embryos at one-cell stage.

### Generation of constitutively active constructs

Constitutively active versions of Smad2, Smad3a, and Smad3b were generated by site-directed mutagenesis of their C-terminal serines to aspartic acids in the SSXS phosphorylation motifs (phosphomimetic mutation)^67^. By using Cold Fusion technology, these constitutively active versions were cloned into a Tol2 vector under the control of *myl7* promoter, *Tg(myl7:H2B-EGFP-2A-caSmad2)*, *Tg(myl7:H2B-EGFP-2A-caSmad3a)*, *Tg(myl7:H2B-EGFP-2A-caSmad3b)*. Similarly, *Tg(myl7:H2B-EGFP)* was generated as control. 20 pg of each of these constructs were co-injected with 20 pg Tol2 mRNA into one-cell stage embryos.

### Morpholinos

The following MOs were purchased from GeneTools (Philomath, OR) and injected at the one-cell stage at the indicated amounts in all experiments described: *acvr2aa* ATG MO—1.5 ng (5′-CCAGCTTTGTTGCAGGTCCCATTTT-3′), *acvr2ab* splice MO—2 ng (5′-TGGCTGCACACAAACACAGATTAAT-3′), *acvr2ba* ATG MO—1 ng (5′-TGAGCAGAGAAGCGAACATATTCCT-3′), *acvr2bb* ATG MO—0.5 ng (5′-AGCCAGCCAGGGAACAAACATATTC-3′) and control MO—concentrations similar to experimental MOs (5′-CCTCTTACCTCAGTTACAATTTATA-3′). All doses were determined as optimal by titration (no toxic effects were observed).

### Histology and in situ hybridization

The hearts were fixed in 4% paraformaldehyde (PFA) and 7 µm thick paraffin sections or 12 µm thick cryosections were obtained. For H&E staining, the cryosections were stained with acidic hemalum (Waldeck) for 10 min, washed in running tap water for 2 min and rinsed in deionized water. Further, the sections were stained with eosin (Waldeck) for 6 min, dehydrated in 100% ethanol, cleared in xylene and mounted in entellan (Merck). For AFOG staining, paraffin sections were fixed with Bouin’s solution overnight at room temperature (RT) and stained according to the manufacturer’s instructions (Gennova), without hematoxylin solution. To perform in situ hybridization^68^, cryosections were permeabilized in 5 μg ml^−1^ proteinase K (Roche) for 15 min at RT, followed by acetylation for 2 min and pre-incubation in hybridization buffer for 3 h at 70 °C. Later, the sections were incubated with DIG-labeled RNA antisense probes overnight at 70 °C. Next, the sections were washed and incubated with alkaline phosphatase-conjugated anti-digoxigenin antibody (Roche) overnight at 4 °C. Finally, after washing, the signal was detected with NBT-BCIP staining solution (Roche). Probes for in situ hybridization were generated by using the following primer sequences: *mstnb_*insitu_F-5′-CCCATTGTTCAAGTAGATCGG-3′, *mstnb_*insitu_R-5′-ATTGTCCATTCCCGAGTCCA-3′, *inhbaa_*insitu_F-5′-ATCATCACGT TCGCTGAAACC-3′ and *inhbaa_*insitu_R-5′-GAGAGTTCGTCTTGAGGCAG-3′.

### Immunofluorescence

To perform immunofluorescence^7^, cryosections were fixed in 4% PFA for 15 min, followed by antigen retrival for 20 min at 95 °C (for PCNA antibody staining), permeabilization in 0.5% Triton-X for 15 min at RT and incubation in blocking buffer (1% BSA in PBS) for 1 h at RT. Later, the sections were incubated in primary antibody overnight at 4 °C. Next, after washing, the sections were incubated with secondary antibody overnight at 4 °C. Finally, the immunostained slides were mounted with mowiol for imaging. To perform immunofluorescence^69^, whole-mount larvae were fixed in 4% PFA overnight at 4 °C, followed by incubation in permeabilization solution (0.3% Triton-X, 1% DMSO, 1% BSA, and 0.1% Tween-20) for 3 h at RT. Further, the larvae were incubated in blocking solution (1% DMSO, 2% FBS, 1% BSA, and 0.1% Tween-20) for 1 h at RT. Next, the larvae were incubated in primary antibody overnight at 4 °C. Later, after washing, the larvae were incubated in secondary antibody overnight at 4 °C. Finally, the stained larvae were washed, mounted in 1.5% low melting agarose for imaging. Primary antibodies used for immunofluorescence were anti-PCNA at 1:200 (mouse; Dako), anti-DsRed at 1:300 (rabbit, Clontech), anti-pSmad3 at 1:200 (rabbit; Abcam), anti-MF-20 at 1:500 (mouse; eBioscience), anti-GFP at 1:500 (chicken, Aves Labs) and anti-N2.261 at 1:50 (mouse, H.M. Blau, Developmental Studies Hybridoma Bank). Secondary antibodies were used at 1:500 (Life Technologies).

### Imaging and quantification

The immunostained slides and larvae were imaged at ×20 magnification and ×40 magnification, respectively using LSM700/LSM800 confocal microscopes (Zeiss). After imaging, the acquired confocal z-stacks were processed and cell counting was performed with ZEN (Zeiss), Fiji, and Imaris (Bitplane) softwares. Bright field images were obtained with stereomicroscopes (SMZ25, Nikon and Stereodiscovery V8, Zeiss). Ventricular, atrial and eye sizes were measured by using ZEN software (apex to base). Ventricular wall thickness was also measured by using ZEN software, by taking the average of three regions near the apex.

### Genotyping

For genotyping the immunostained larvae obtained by crossing different transgenic backgrounds, PCR was performed on genomic DNA using the primers listed in Supplementary Table 4. *erbb2* ^*st61*^ mutants were genotyped^63^ by using PCR-based restriction fragment length polymorphism analysis. PCR products obtained from genomic DNA samples (using primer pairs listed in Supplementary Table 4) were cut with BsrGI, resulting in a genotype-specific DNA band pattern.

### EdU treatment

For EdU incorporation analysis, adult fish were anaesthetized with 0.016% tricaine and 200 µg of EdU (Invitrogen) was injected intraperitoneally. The hearts were sampled after 3 days of EdU incubation and fixed in 4% PFA. 1 mM EdU was used to incubate embryos from 48 hpf to 72 hpf and larvae from 96 hpf to 120 hpf, followed by fixation in 4% PFA. EdU labeling was performed according to the manufacturer’s protocol (Invitrogen).

### ErBb2 and TGF-β signaling inhibitor treatments

The ErBb2 signaling inhibitor (PD168393, Calbiochem)^70^, Smad3 phosphorylation inhibitor (SIS3, Calbiochem)^50^ and Activin type 1 receptor inhibitor (SB431542, Calbiochem)^52^ were used to treat the embryos or larvae. The embryos were treated with 3 µM SIS3 and 10 µM SB431542 from 36 hpf to 72 hpf. The larvae were treated with 10 µM PD168393 and 3 µM SIS3 from 84 hpf to 120 hpf. All inhibitors were dissolved in DMSO and added to egg water. Control fish were incubated in 1% DMSO in egg water.

### Statistical analysis

No statistical methods were used to predetermine sample size. The experiments were not randomized. The investigators were not blinded to allocation during experiments or outcome assessment, except for the data shown in Fig. 4e–s. GraphPad software was used to perform statistical analysis. Data are represented as mean ± s.e.m. *P*-values were calculated by two-tailed Student’s *t* test.

### Data availability

The authors declare that all data supporting the findings of this study are available within the article and its Supplementary Information files or from the corresponding author upon reasonable request. Microarray raw and normalized data have been deposited in the NCBI-GEO database under the accession code: GSE89259.

## Electronic supplementary material

Supplementary Information
