# Supplementary material for: Opposite effects of Activin type 2 receptor ligands on cardiomyocyte proliferation during development and repair
Source: Nat Commun. 2017 Dec 1;8:1902. doi: 10.1038/s41467-017-01950-1 (PMC5711791; doi:10.1038/s41467-017-01950-1)
Supplement: Supplementary file 1 — Supplementary Information [file 41467_2017_1950_MOESM1_ESM.pdf]

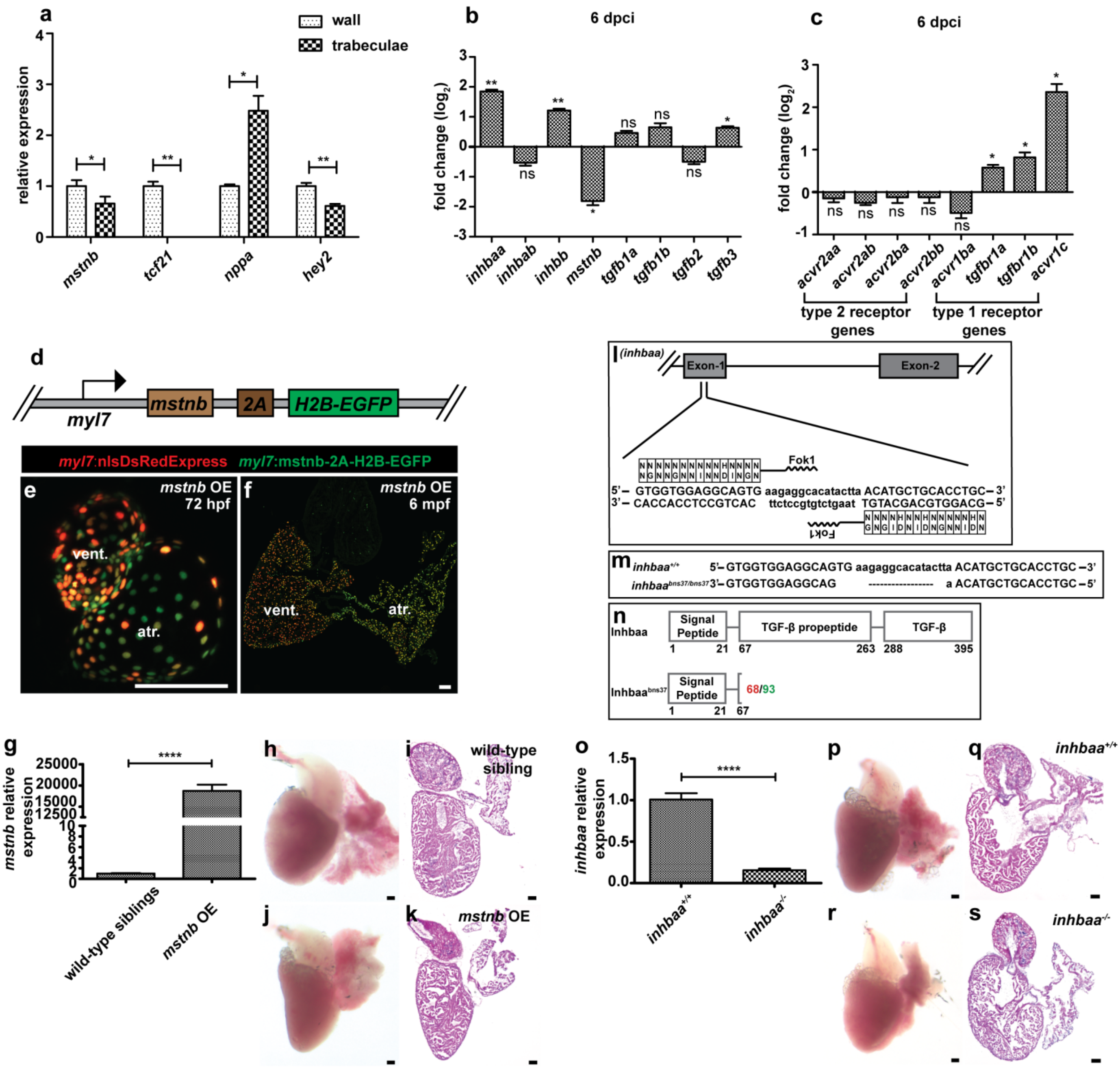

Supplementary Figure 1. Expression analysis for TGF-β family genes and generation of *mstnb* GOF and *inhbaa* LOF lines. (**a**) RT–qPCR for *mstnb*, *tcf21* (wall-specific), *nppa* (trabecular-specific) and *hey2* (wall-specific) expression in Laser micro dissected wall and trabecular tissues from adult zebrafish heart sections (n=3 hearts assessed in 2 technical replicates). (**b**) RT–qPCR for TGF-β family ligand gene expression at 6 dpci compared to sham injured hearts (n=2 x 3 cardiac ventricles assessed as 2 biological and 2 technical replicates). (**c**) RT–qPCR for TGF-β family receptor gene expression at 6 dpci compared to sham injured hearts (n=2 x 3 cardiac ventricles assessed as 2 biological and 2 technical replicates). (**d**) Schematic representation of CM-specific *mstnb* OE transgene,

Tg(myl7:mstnb-2A-H2B-EGFP). (**e**) Heart of 72 hpf *mstnb* OE larva in *Tg(myl7:nlsDsRedExpress)* background (native fluorescence). (**f**) Section of *mstnb* OE adult heart in *Tg(myl7:nlsDsRedExpress)* background; α-DsRed (red), α-GFP (green). (**g**) RT–qPCR for *mstnb* expression analysis in wild-type sibling and *mstnb* OE adult hearts (n=2 x 3 cardiac ventricles assessed as 2 biological and 2 technical replicates). (**h- k**) *mstnb* OE adult hearts and Hematoxylin and Eosin (H&E) staining of heart sections show no apparent abnormalities. (**l-n**) *inhbaa* TALEN designed to target TGF-β propeptide domain encoded by exon 1 was generated (**l**), a 17 bp frameshift deletion ($inhbaa^{bns37}$) was identified (**m**) and is predicted to cause the formation of truncated Inhbaa (**n**). Red number indicates the last native amino acid before the frameshift mutation; green number indicates the last amino acid before stop codon. (**o**) RT–qPCR for *inhbaa* expression analysis in $inhbaa^{+/+}$ and $inhbaa^{-/-}$ adult hearts (n=2 x 3 cardiac ventricles assessed as 2 biological and 2 technical replicates). (**p-s**) $inhbaa^{-/-}$ adult hearts and H&E staining of heart sections show no apparent abnormalities. (data are mean ± s.e.m., ns: no significant changes observed, *P ≤ 0.05, **P ≤ 0.01 and ****P ≤ 0.0001 - Student's t-test, two-tailed). Scale bars, 100 µm. vent., ventricle; atr., atrium; dpf, days post fertilization; mpf, months post fertilization.

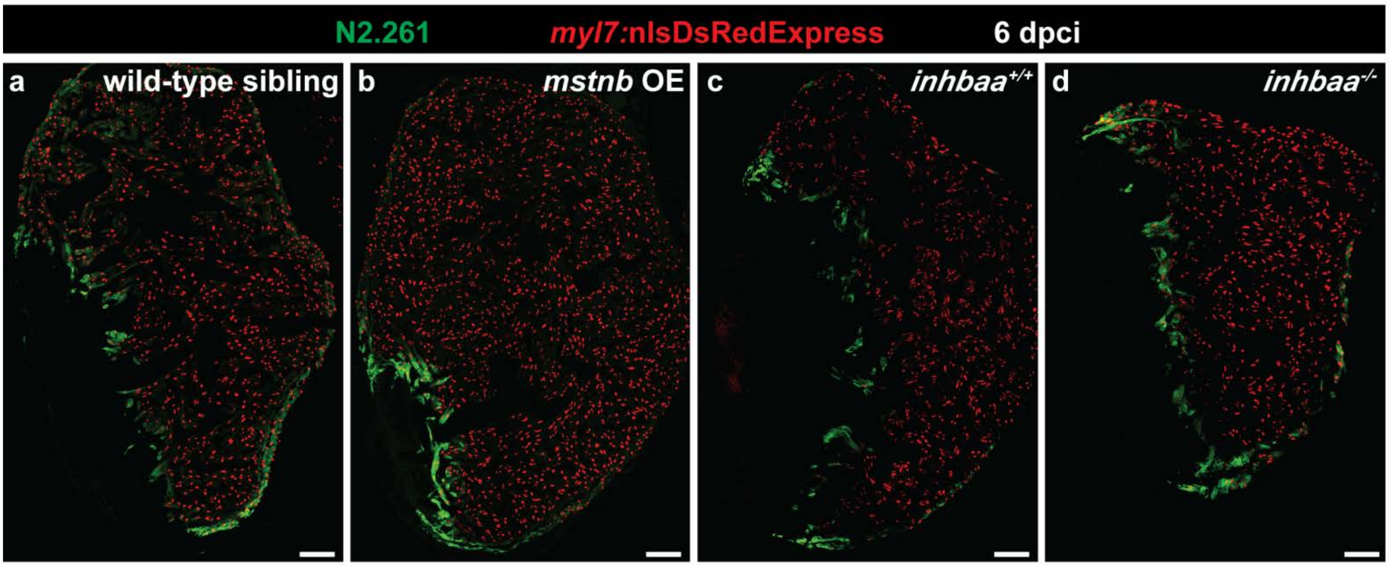

Supplementary Figure 2. ***mstnb*** **GOF and** ***inhbaa*** **LOF do not affect CM dedifferentiation during cardiac regeneration.** (**a-d**) Sections of wild-type sibling (n=3), *mstnb* OE (n=3), *inhbaa*$^{+/+}$ (n=3) and *inhbaa*$^{-/-}$ (n=3) cryoinjured hearts in *Tg(myl7:nlsDsRedExpress)* background at 6 dpci; α-DsRed (red), α-N2.261 (green). Scale bars, 100 µm.

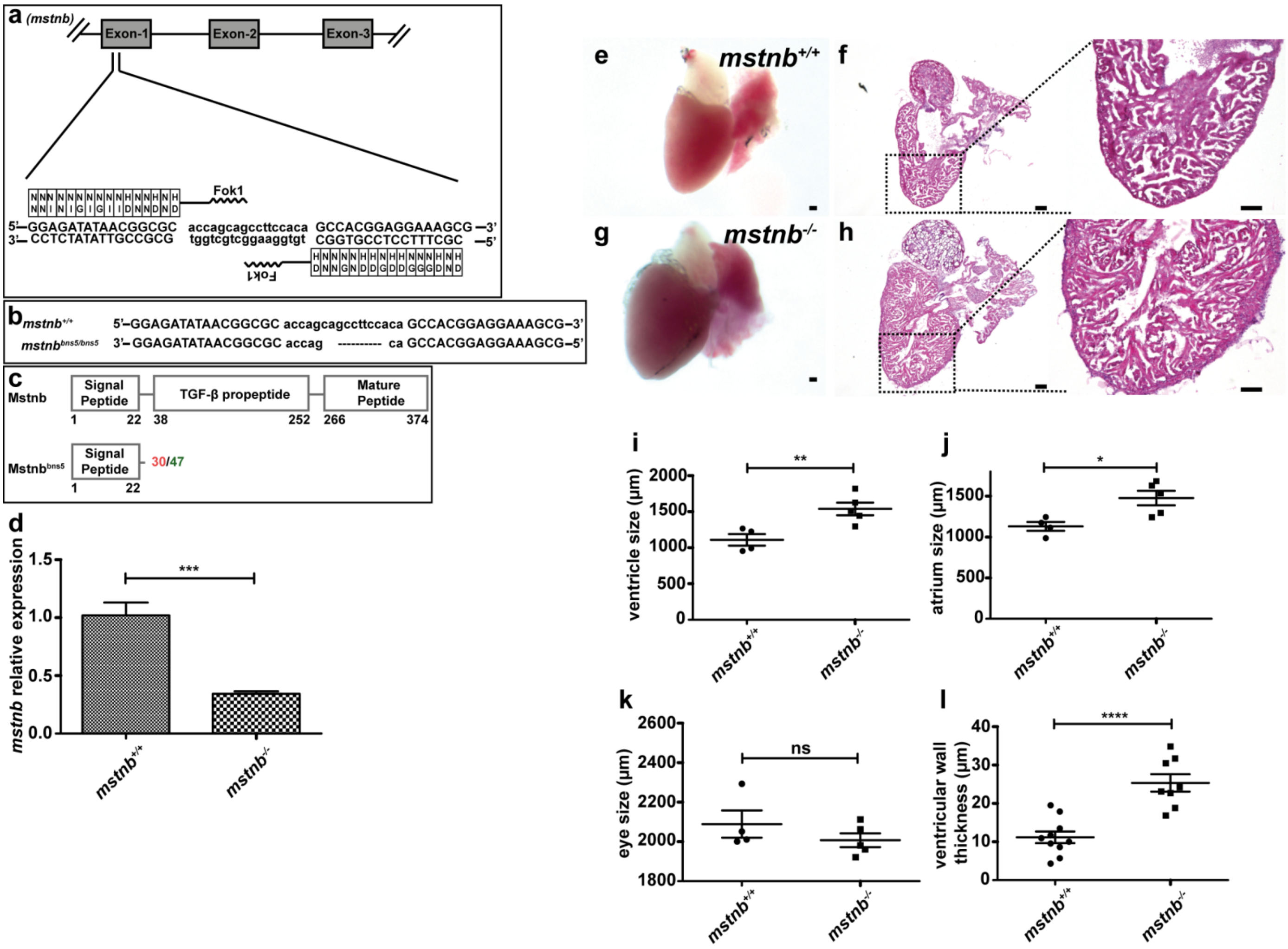

Supplementary Figure 3. Generation and validation of *mstnb* LOF line. (**a-c**) *mstnb* TALEN designed to target the region after the signal peptide encoded by exon 1 was generated (**a**), a 10 bp frameshift deletion (*mstnb*^{bns5}) was identified (**b**) and is predicted to cause the formation of truncated Mstnb (**c**). Red number indicates the last native amino acid before the frameshift mutation; green number indicates the last amino acid before stop codon. (**d**) RT–qPCR for *mstnb* expression analysis in *mstnb*$^{+/+}$ and *mstnb*$^{-/-}$ adult hearts (n=2 x 3 cardiac ventricles assessed as 2 biological and 2 technical replicates). (**e-h**) *mstnb*$^{+/+}$ and *mstnb*$^{-/-}$ adult hearts, H&E staining of heart sections and higher magnifications of H&E staining showing representative enlarged heart with thickened ventricular wall in *mstnb*$^{-/-}$ animals. (**i-k**) Quantification of ventricle, atrium and eye size in *mstnb*$^{+/+}$ (n=4) and *mstnb*$^{-/-}$ (n=5) adults. (**l**) Quantification of ventricular wall thickness in *mstnb*$^{+/+}$ (n=10) and *mstnb*$^{-/-}$ (n=8) adult hearts. (data are mean ± s.e.m., ns: no significant changes observed, *P ≤ 0.05, **P ≤ 0.01, ***P ≤ 0.001 and ****P ≤ 0.0001 - Student's t-test, two-tailed). Scale bars, 100 μm.

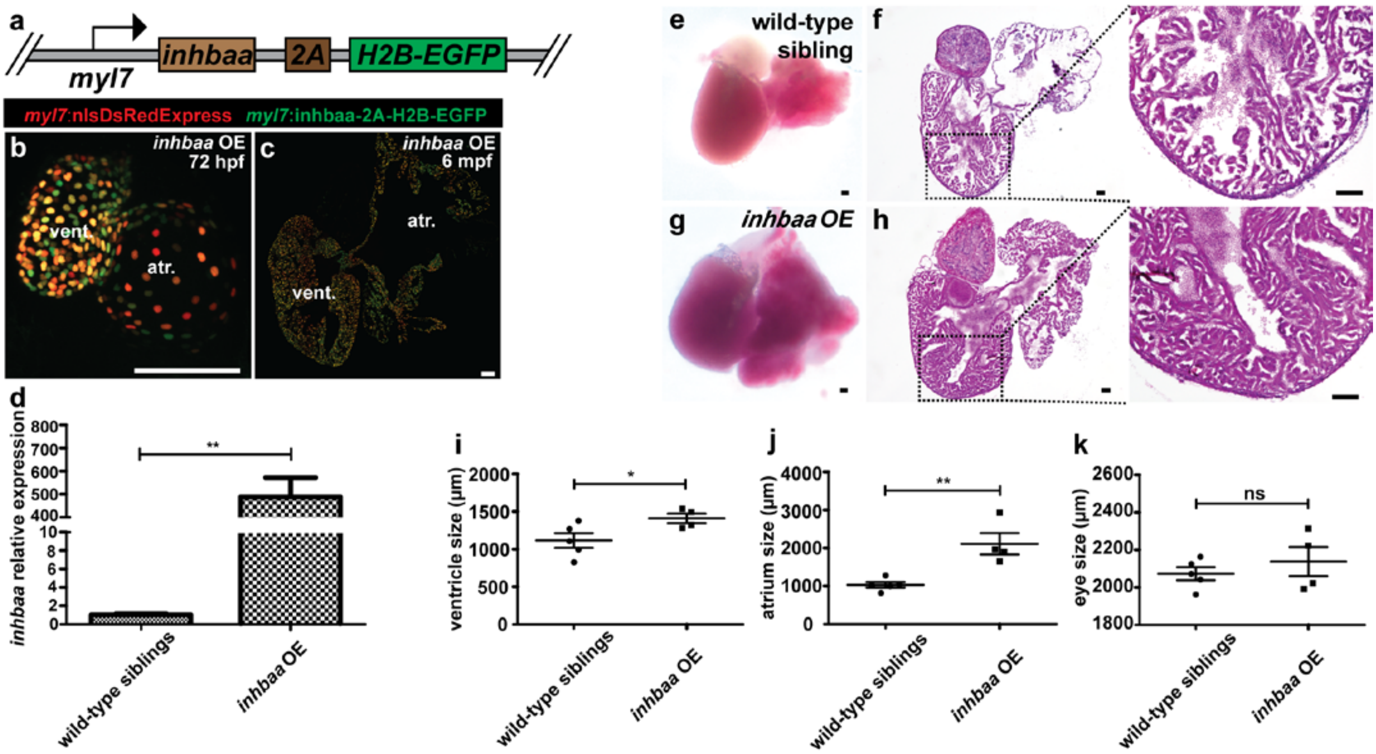

Supplementary Figure 4. Generation and validation of *inhbaa* GOF line. (**a**) Schematic representation of CM-specific *inhbaa* OE transgene, *Tg(myl7:inhbaa-2A-H2B-EGFP).* (**b**) Heart of 72 hpf *inhbaa* OE larva in *Tg(myl7:nlsDsRedExpress)* background (native fluorescence). (**c**) Section of *inhbaa* OE adult fish heart in *Tg(myl7:nlsDsRedExpress)* background; α-DsRed (red), α-GFP (green). (**d**) RT–qPCR for *inhbaa* expression analysis in wild-type sibling and *inhbaa* OE adult hearts (n=2 x 3 cardiac ventricles assessed as 2 biological and 2 technical replicates). (**e-h**) Wild-type sibling and *inhbaa* OE adult hearts, H&E staining of heart sections and higher magnifications of H&E staining showing representative enlarged heart with dense trabeculae in *inhbaa* OE animals. (**i-k**) Quantification of ventricle, atrium and eye size in wild-type sibling (n=5) and *inhbaa* OE (n=4) adults. (data are mean ± s.e.m., ns: no significant changes observed, *P ≤ 0.05 and **P ≤ 0.0 by Student's t-test, two-tailed). Scale bars, 100 μm. vent., ventricle; atr., atrium.

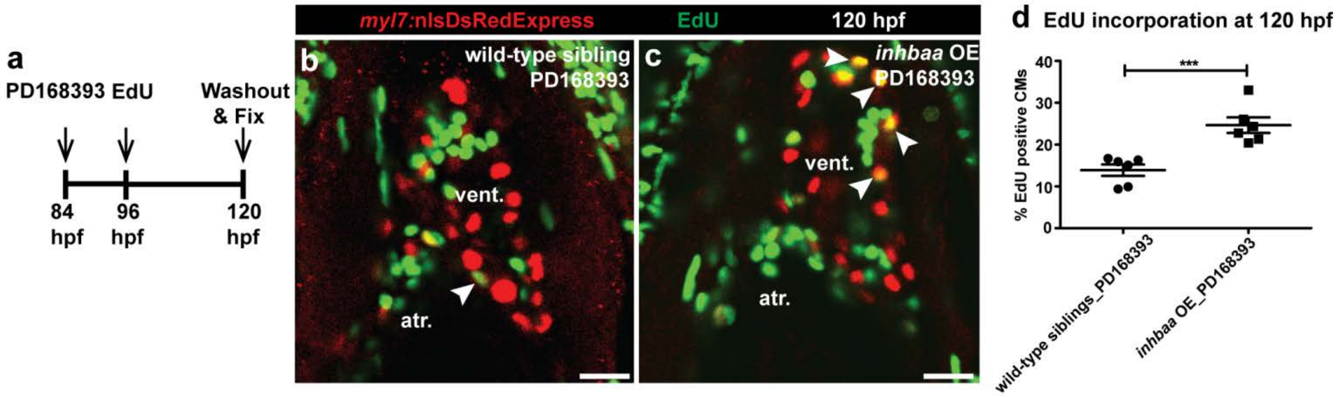

Supplementary Figure 5. ***inhbaa*** **GOF promotes CM proliferation during ErbB2 signaling inhibition.** (**a**) Experimental setup of PD168393 treatment, EdU exposure, followed by fixation. (**b, c**) *Tg(myl7:nlsDsRedExpress)* hearts of PD168393-treated wild-type sibling and *inhbaa* OE larvae at 120 hpf; α-DsRed (red), EdU (green). White arrowheads point to proliferating CMs (EdU^+/$DsRed^+$). (**d**) Quantification of CM proliferation in PD168393-treated wild-type sibling (n=6) and *inhbaa* OE (n=6) ventricles at 120 hpf. All cell counts were performed on non-overlapping confocal planes (thickness, 1µm) (data are mean ± s.e.m., ***$P \leq 0.001$ - Student's t-test, two-tailed). Scale bars, 20 µm. vent., ventricle; atr., atrium.

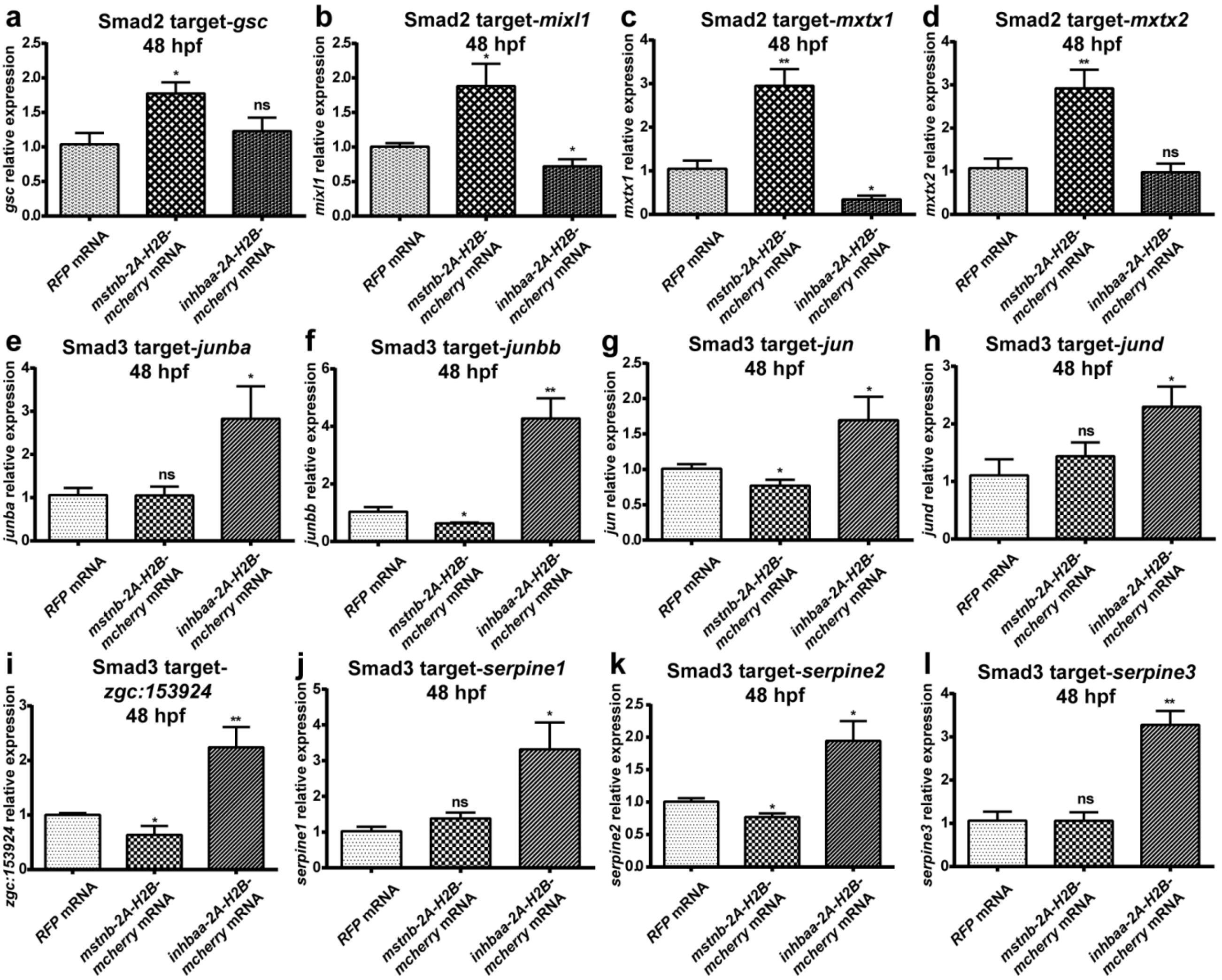

Supplementary Figure 6. ***mstnb*** **and** ***inhbaa*** **inversely regulate Smad2 and Smad3 activities during early embryonic development.** (**a-d**) RT-qPCR analysis for Smad2 target gene expression in 48 hpf embryos injected with *mstnb-2A-H2B-mcherry* mRNA or *inhbaa-2A-H2B-mcherry* mRNA compared to *RFP* mRNA injected (n=2 x 10 embryos assessed as 2 biological and 2 technical replicates). (**e-l**) RT-qPCR analysis for Smad3 target gene expression in 48 hpf embryos injected with *mstnb-2A-H2B-mcherry* mRNA or *inhbaa-2A-H2B-mcherry* mRNA compared to *RFP* mRNA injected (n=2 x 10 embryos assessed as 2 biological and 2 technical replicates) (data are mean ± s.e.m., ns: no significant changes observed, *P ≤ 0.05 and **P ≤ 0.01- Student’s t-test, two-tailed).

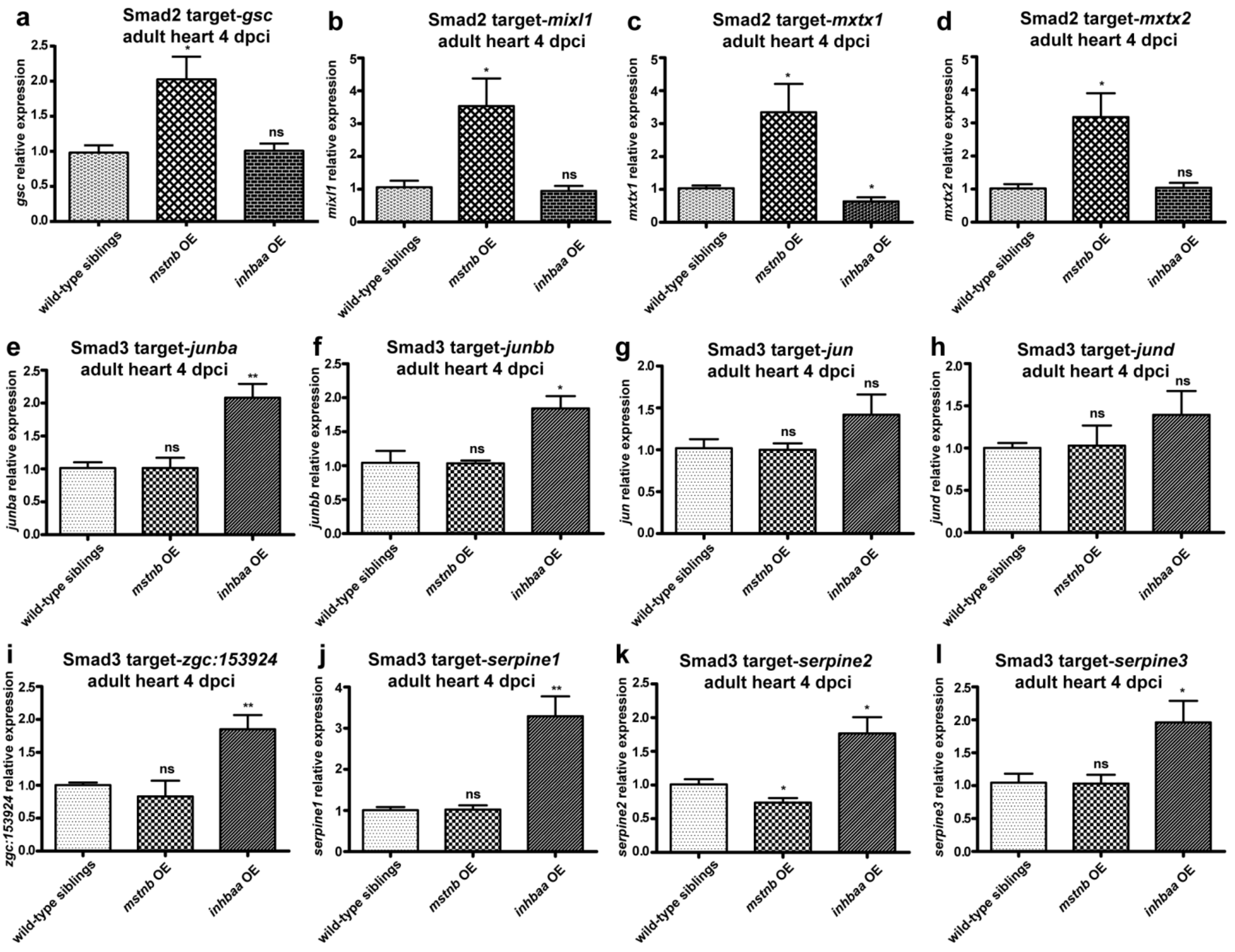

Supplementary Figure 7. ***mstnb*** **and** ***inhbaa*** **inversely regulate Smad2 and Smad3 activities in injured adult heart.** (**a-d**) RT-qPCR analysis for Smad2 target gene expression in *mstnb* OE and *inhbaa* OE 4 dpci adult hearts compared to 4 dpci wild-type hearts (n=2 x 3 cardiac ventricles assessed as 2 biological and 2 technical replicates). (**e-l**) RT-qPCR analysis for Smad3 target gene expression in *mstnb* OE and *inhbaa* OE 4 dpci adult hearts compared to 4 dpci wild-type hearts (n=2 x 3 cardiac ventricles assessed as 2 biological and 2 technical replicates) (data are mean ± s.e.m., ns: no significant changes observed, *P ≤ 0.05 and **P ≤ 0.01- Student's t-test, two-tailed).

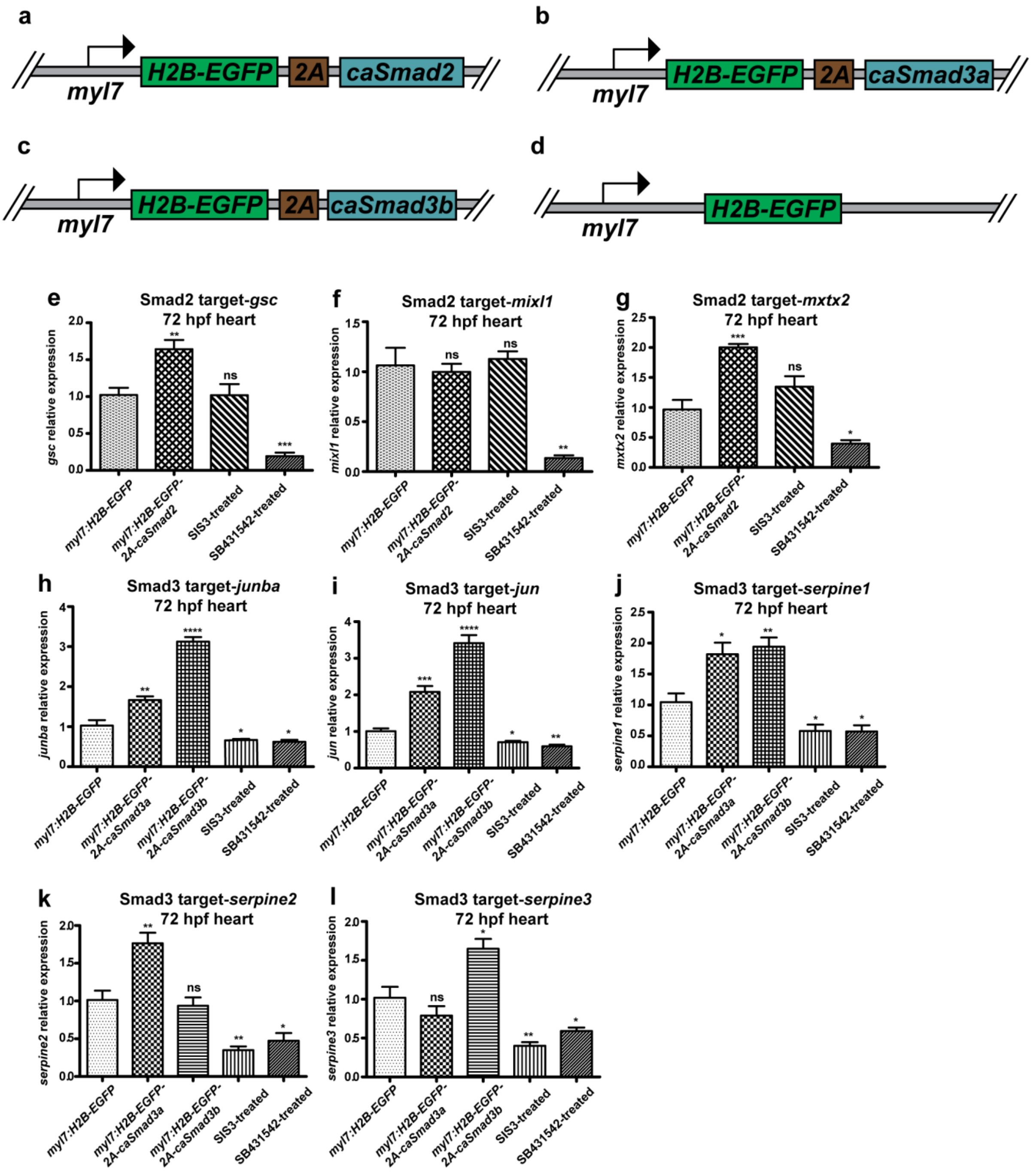

Supplementary Figure 8. Validation of generated caSmad constructs and TGF-β signaling inhibitors. (**a-d**) Schematic representation of CM-specific constructs, *Tg(myl7:H2B-EGFP-2A-caSmad2)*, *Tg(myl7:H2B-EGFP-2A-caSmad3a)*, *Tg(myl7:H2B-EGFP-2A-caSmad3b)* and *Tg(myl7:H2B-EGFP)*. (**e-g**) RT-qPCR analysis for Smad2 target gene expression in *myl7:H2B-GFP*, *caSmad2*, SIS3-treated and SB431542-treated 72 hpf hearts (n=2 x 30 hearts assessed as 2 biological and 2 technical replicates). (**h-l**) RT-qPCR analysis for Smad3 target gene expression in *myl7:H2B-GFP*, *caSmad3a*, *caSmad3b*, SIS3-treated and SB431542-treated 72 hpf hearts (n=2 x 30 hearts assessed as 2 biological and 2

technical replicates) (data are mean ± s.e.m., ns: no significant changes observed, *$P \leq 0.05$, **$P \leq 0.01$, ***$P \leq 0.001$ and ****$P \leq 0.0001$ - Student's t-test, two-tailed).

Supplementary Table 1. Internal gene names used and their corresponding ensemble names

Internal gene name	Ensemble gene name	Ensemble gene ID
acvr2ba	*acvr2b*	ENSDARG00000044422
acvr2bb	*ACVR2B*	ENSDARG00000103108

Supplementary Table 2. List of primers used for RT-qPCR

Primer names	Primer sequences
*mstnb*_qpcr_F	5’-CGTCACTTCAACCGAGACTG-3’
*mstnb*_qpcr_R	5’-ATTCTCCTGAACAGTAATTCGCC-3’
*inhbaa*_qpcr_F	5’-AGAACAGACGGAGATCATCAC-3’
*inhbaa*_qpcr_R	5’-GCAGTCGAAGGAAGATCCAG-3’
*inhbab*_qpcr_F	5’-GTCAAGCAGCACATCCTCAA-3’
*inhbab*_qpcr_R	5’-CCTCCGTCTTCCTCCATCTC-3’
*inhbb*_qpcr_F	5’-ACCAAATATCACTCATCCCATTCC-3’
*inhbb*_qpcr_R	5’-TGGAGTTACATCATCTGATTCGG-3’
*tgfb1a*_qpcr_F	5’-TTCCAGCAAGCTCAGAATAACAC-3’
*tgfb1a*_qpcr_R	5’-TGTCTGTTTCACGTCAAATGAGAG-3’
*tgfb1b*_qpcr_F	5’-CATTGACTTCCGCAAAGACC-3’
*tgfb1b*_qpcr_R	5’-ACAACTGTTCCACCTTATGCT-3’
*tgfb2*_qpcr_F	5’-CTACACAGAGACAGAAACAAAGG-3’
*tgfb2*_qpcr_R	5’-GAGCAGAAAGCAGTATCCAG-3’
*tgfb3*_qpcr_F	5’-CAAACAGAGCAACGAACCTG-3’
*tgfb3*_qpcr_R	5’-TGAGAAGCAGTATTTGGTGTC-3’
*acvr2aa*_qpcr_F	5’-CCAGTTCAGACGACATCAAACC-3’
*acvr2aa*_qpcr_R	5’-GTATGCCAACTTGTGATGCC-3’
*acvr2ab*_qpcr_F	5’-CCATGAGAAGGGTTCACTGAC-3’
*acvr2ab*_qpcr_R	5’-TAGATCACTCTTCAGCAGCAC-3’
*acvr2ba*_qpcr_F	5’-GACTCCTTTCTGCGGATAGAC-3’
*acvr2ba*_qpcr_R	5’-GACACAGACCTGAATGCTTGAG-3’
*acvr2bb*_qpcr_F	5’-CTTCTGATGGTCCTGTAGGT-3’
*acvr2bb*_qpcr_R	5’-TAAACCCTGATGCTTGACCC-3’
*acvr1ba*_qpcr_F	5’-ACTGATACTATAGACATTGCACCC-3’
*acvr1ba*_qpcr_R	5’-CATGGATACCTCCAGCATTACAC-3’
*tgfbr1a*_qpcr_F	5’-AAAGTTATCCCAGAGTTGCCT-3’
*tgfbr1a*_qpcr_R	5’-ACACGATGGTGGATGATTGAG-3’
*tgfbr1b*_qpcr_F	5’-GACATCGCTCCCAATCACAG-3’
*tgfbr1b*_qpcr_R	5’-TCATGAATACCTCCGATAGAGCAG-3’
*acvr1c*_qpcr_F	5’-TCAATCCAGAGCAACCACCT-3’

*acvr1c*_qpcr_R	5’-CACACTCCTAGCATGACACC-3’
*nppa*_qpcr_F	5’-ACGCATTCAGAGACACTCAG-3’
*nppa*_qpcr_R	5’-TTGCTGTCTTCATAATCTACGG-3’
*tcf21*_qpcr_F	5’-TCAGAATGTACAGGAAAGAGGC-3’
*tcf21*_qpcr_R	5’-CTTGGAGAGTTTGGTGTCCG-3’
*hey2*_qpcr_F	5’-GCAGCGAGAATAACTACTCTGG-3’
*hey2*_qpcr_R	5’-TTTCAATGATCCCTCTCCGCT-3’
*EGFP*_qpcr_F	5’-AAGCTGACCCTGAAGTTCATCTGC-3’
*EGFP*_qpcr_R	5’-CTTGTAGTTGCCGTCGTCCTTGAA-3’
*junba*_qpcr_F	5’-GCAATCCTAACACTAACCTCACAC-3’
*junba*_qpcr_R	5’-TCCTCTTTCAGAGTAACGAGCC-3’
*junbb*_qpcr_F	5’-CCACAAGATGAACCAGATGCC-3’
*junbb*_qpcr_R	5’-TTCAGCGTCGTGTAAATGGG-3’
*jun*_qpcr_F	5’-GGAAACTACTTTCTACGATGACTC-3’
*jun*_qpcr_R	5’-CTAGACTGGATGATGAGCCT-3’
*jund*_qpcr_F	5’-GCTTCGCATAAACAAGGATATACG-3’
*jund*_qpcr_R	5’-GTTGAGTATTCCCTCAGCATCC-3’
*zgc:153924*_qpcr_F	5’-GACGGGTAAGATGGAAACGC-3’
*zgc:153924*_qpcr_R	5’-TGCTCTTCTTCATCATCTTGTGG-3’
*serpine1*_qpcr_F	5’-CACACTGATGGAATGATTTCTGAG-3’
*serpine1*_qpcr_R	5’-CATCCTTAGACACGAACTCAC-3’
*serpine2*_qpcr_F	5’-GCACTAAAGGCCAGATTCCC-3’
*serpine2*_qpcr_R	5’-GCACTTTGTATGTGTTTCCGTC-3’
*serpine3*_qpcr_F	5’-AACTCCTGCCAGAATTTACCC-3’
*serpine3*_qpcr_R	5’-TGGTCATCAGCTTTACTTTGG-3’
*gsc*_qpcr_F	5’-TGTGCTTATTTCTCCAGTCCC-3’
*gsc*_qpcr_R	5’-TGAACCAAACCTCTACCTTCTC-3’
*mixl1*_qpcr_F	5’-GACAACGTCAGTTTGTTTACAC-3’
*mixl1*_qpcr_R	5’-GGAACCAGACCTGAATTCTC-3’
mxtx1_qpcr_F	5’-CTGCGAGCTACATTTGAAACAG-3’
mxtx1_qpcr_R	5’-GAACCAGACCTGTATGCGAG-3’
mxtx2_qpcr_F	5’-CACGTATTCAGGTATGGTTCCAG-3’
mxtx2_qpcr_R	5’-TGGATGTTGAAGGGTGACTC-3’
*rpl13*_qpcr_F	5’-TAAGGACGGAGTGAACAACCA-3’
*rpl13*_qpcr_R	5’-CTTACGTCTGCGGATCTTTCTG-3’

Supplementary Table 3. **Ct values of genes in RT-qPCR**

Figures	Genes	Ct values in control
Fig. 1c	*mstnb*	25.60
Fig. 1d	*inhbaa*	27.80
Fig. 5a	*EGFP*	25.51
Fig. 5b	*EGFP*	20.33
Fig. 7a, c, e, g	*EGFP*	24.29
Fig. 7b, d, g, h	*EGFP*	19.55
Supplementary Fig. 1a	*mstnb*	23.90
Supplementary Fig. 1a	*tcf21*	22.07
Supplementary Fig. 1a	*nppa*	17.00
Supplementary Fig. 1a	*hey2*	22.80
Supplementary Fig. 1b	*inhbaa*	27.53
Supplementary Fig. 1b	*inhbab*	26.51
Supplementary Fig. 1b	*inhbb*	24.46
Supplementary Fig. 1b	*mstnb*	25.22
Supplementary Fig. 1b	*tgfb1a*	23.32
Supplementary Fig. 1b	*tgfb1b*	23.73
Supplementary Fig. 1b	*tgfb2*	19.00
Supplementary Fig. 1b	*tgfb3*	20.27
Supplementary Fig. 1c	*acvr2aa*	22.54
Supplementary Fig. 1c	*acvr2ab*	22.17
Supplementary Fig. 1c	*acvr2ba*	23.74
Supplementary Fig. 1c	*acvr2bb*	24.14
Supplementary Fig. 1c	*acvr1ba*	21.35
Supplementary Fig. 1c	*tgfbr1a*	25.27
Supplementary Fig. 1c	*tgfbr1b*	23.03
Supplementary Fig. 1c	*acvr1c*	26.60
Supplementary Fig. 1g	*mstnb*	25.68
Supplementary Fig. 1m	*inhbaa*	27.88
Supplementary Fig. 3d	*mstnb*	25.94
Supplementary Fig. 4d	*inhbaa*	27.01
Supplementary Fig. 6a	*gsc*	23.19
Supplementary Fig. 6b	*mixl1*	28.99
Supplementary Fig. 6c	*mxtx1*	30.39
Supplementary Fig. 6d	*mxtx2*	30.27
Supplementary Fig. 6e	*junba*	26.26
Supplementary Fig. 6f	*junbb*	28.59
Supplementary Fig. 6g	*jun*	20.34
Supplementary Fig. 6h	*jund*	22.82
Supplementary Fig. 6i	*zgc:153924*	25.52
Supplementary Fig. 6j	*serpine 1*	24.38
Supplementary Fig. 6k	*serpine 2*	22.25
Supplementary Fig. 6l	*serpine3*	29.56
Supplementary Fig. 7a	*gsc*	28.68

Supplementary Fig. 7b	*mixl1*	32.55
Supplementary Fig. 7c	*mxtx1*	33.94
Supplementary Fig. 7d	*mxtx2*	31.32
Supplementary Fig. 7e	*junba*	22.77
Supplementary Fig. 7f	*junbb*	28.16
Supplementary Fig. 7g	*jun*	19.96
Supplementary Fig. 7h	*jund*	20.15
Supplementary Fig. 7i	*zgc:153924*	23.22
Supplementary Fig. 7j	*serpine 1*	20
Supplementary Fig. 7k	*serpine 2*	22.44
Supplementary Fig. 7l	*serpine3*	25.73
Supplementary Fig. 8e	*gsc*	26.48
Supplementary Fig. 8f	*mixl1*	30.93
Supplementary Fig. 8g	*mxtx2*	32.72
Supplementary Fig. 8h	*junba*	26.01
Supplementary Fig. 8i	*jun*	24.15
Supplementary Fig. 8j	*serpine 1*	29.07
Supplementary Fig. 8k	*serpine 2*	27.97
Supplementary Fig. 8l	*serpine3*	28.59

Supplementary Table 4. List of primers used for genotyping

**Primer names**	**Primer sequences**
mstnb OE_PCR_F	5’-GAGGACTTTGGCTGGGACTG-3’
mstnb OE_PCR_R	5’-ATGGCCTTAGTACCCTCGGAC-3’
inhbaa OE_PCR_F	5’-TCCATGCTCTACTACAACGAGGAG-3’
inhbaa OE_PCR_R	5’-ATGGCCTTAGTACCCTCGGAC-3’
nrg2a OE_PCR_F	5’-CAGGTGCCTACCTTCCAGATCAC-3’
nrg2a OE_PCR_R	5’-GCAGCCCATACTAGTAGGTCC-3’
$erbb2^{st61}$ _PCR_F	5’-TGAAGAATGCTGGTAGCTGG-3’
$erbb2^{st61}$ _PCR_R	5’-GGACTCAGCAAAGGACTTAC-3’
